# Role of SLC16A10 in Psoriasis Through the Regulation of Arachidonic Acid Metabolism in Keratinocytes

**DOI:** 10.1002/advs.202417093

**Published:** 2025-09-08

**Authors:** Jingyuan Yang, Yixuan Chen, Bowei Li, Shuang Wu, Hang Liang, Xingyue Yang, Xiaozhen Li, Ping Sun, Guangjin Guo, Ting Li, Yuying Jia, Haijiao Li, Mengqi Bai, Jie Xu, Zhijie Liu, Wei Liu, Xiangkang Jiang, Hong Cai

**Affiliations:** ^1^ Department of Dermatology Air Force Medical Center PLA Beijing 100142 China; ^2^ China Medical University Shenyang Liaoning 110004 China; ^3^ Chongqing Medical University School of Basic Medicine Chongqing 400016 China; ^4^ Department of Graduate Hebei North University Zhangjiakou Hebei 075000 China; ^5^ Clinical Medicine Laboratory of Air Force Medical Center PLA Beijing 100142 China; ^6^ Department of Plastic and Cosmetic Surgery Daping Hospital Army Medical University Chongqing 400042 China

**Keywords:** arachidonic acid metabolism, guttate psoriasis, keratinocytes, psoriasis, single‐cell RNA sequencing, SLC16A10

## Abstract

Psoriasis is an inflammatory dermatological condition challenging to treat and prone to recurrence. The pathogenesis of psoriasis is closely associated with metabolic disorders, while therapies targeting the dysregulated metabolism in psoriasis remain limited. Therefore, exploring the pathogenesis of psoriasis and identifying potential metabolic therapeutic targets is imperative. In this study, potential biomarkers for clinically targeted metabolic therapies in patients with psoriasis are aimed to be identified. RNA‐sequencing analysis is performed on metabolism‐related genes to identify differentially expressed metabolism‐related genes. Then, various bioinformatics analyses and comprehensive functional experiments are conducted to verify the roles of the identified genes. A key gene *SLC16A10* is identified with significant diagnostic and therapeutic potential for psoriasis. *SLC16A10* is likely involved in arachidonic acid metabolism in keratinocytes through regulating thyroid hormone homeostasis, contributing to the development of psoriasis. *SLC16A10* represents a promising therapeutic target for guttate psoriasis and can improve the outcomes of existing immune‐targeted therapeutic agents. Comprehensive in vitro and in vivo experiments confirm that *SLC16A10* downregulation alleviates the severity of psoriasis and hyperinflammation. Moreover, *SLC16A10* may induce one of the sequelae of psoriasis, namely post‐inflammatory hypopigmentation, by inhibiting melanogenesis. These findings demonstrate the potential of SLC16A10 as a diagnostic biomarker and therapeutic target for psoriasis.

## Graphical abstract

This study demonstrates that SLC16A10 plays a crucial role in psoriasis pathogenesis through arachidonic acid metabolism regulation in keratinocytes. Using multi‐omics analysis and experimental validation, researchers identify SLC16A10 as both a diagnostic biomarker and therapeutic target. Knockdown experiments significantly reduce psoriasis severity while revealing potential involvement in postinflammatory hypopigmentation through melanogenesis inhibition in melanocytes.

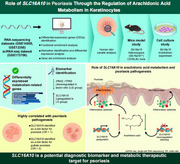

## 1 Introduction

Psoriasis, a common chronic inflammatory skin disease, exhibits a complex etiology involving genetic susceptibility, environmental triggers, and immune dysregulation.^[^ ^1^, ^2^ ^]^ Psoriasis affects ≈2–3% of the population and is accompanied by comorbidities such as cardiovascular disease, metabolic syndrome, psoriatic arthritis, depression, and anxiety.^[^ ^1^ ^]^ Moreover, the quality of life among individuals with psoriasis is further diminished by the severe side effects of clinical treatments and the high recurrence rate after treatment cessation.^[^ ^1^, ^3^ ^]^

The pathophysiological mechanisms underlying psoriasis involve complex interactions between the innate and adaptive immune systems, including inflammation, dysregulated immune responses, uncontrolled proliferation of keratinocytes (KCs), and angiogenesis.^[^ ^3^ ^]^ These processes are orchestrated through intricate crosstalk between extracellular cytokine pathways and intracellular signaling molecules.^[^ ^4^ ^]^ Notably, several multi‐omics approaches have recently been applied in psoriasis research, enhancing our understanding of its genetic structure and pathogenesis.^[^ ^5^, ^6^, ^7^ ^]^ These studies have also identified reliable biomarkers, facilitating the development of targeted therapies. Multiple targeted therapies have been developed and approved for clinical use, including interleukin‐17 (IL‐17) and IL‐23 inhibitors,^[^ ^8^ ^]^ with their efficacy well‐documented. However, these treatments, which target the immunopathogenesis of psoriasis, can cause certain adverse effects.^[^ ^8^, ^9^, ^10^ ^]^ For instance, the most commonly utilized psoriasis‐targeted therapy agents, IL‐17 inhibitors, may induce typical adverse reactions, including upper respiratory tract and injection‐site reactions, and serious adverse reactions, including inflammatory bowel disease and candidiasis.^[^ ^9^, ^10^ ^]^ Furthermore, existing psoriasis‐targeted therapies primarily target plaque‐type psoriasis, and their efficacy and safety profiles for other psoriasis subtypes and comorbidities remain uncertain.^[^ ^10^ ^]^

Recent studies have highlighted the potential of metabolism‐targeted therapies in enhancing the efficacy of immunotherapy for psoriasis.^[^ ^11^, ^12^, ^13^ ^]^ Metabolomic research has identified metabolism as an essential factor in the pathogenesis of psoriasis, demonstrating that various lipid metabolites contribute to its onset.^[^ ^11^, ^14^ ^]^ A hallmark of psoriasis is KC hyperproliferation, which requires high energy demand, thus contributing to the metabolic disruption in psoriasis.^[^ ^15^ ^]^ KCs, as well as associated immune cells, are regulated by various cellular metabolic pathways, including glycolysis, the tricarboxylic acid cycle, lipid metabolism, and amino acid metabolism.^[^ ^11^ ^]^ However, metabolism‐targeted therapies for psoriasis remain underexplored. Therefore, further exploration into the pathogenesis of psoriasis and the identification of potential biomarkers through metabolic pathways, particularly in KCs, could provide new insights into the disease. Such research may facilitate early diagnosis and intervention, leading to advances in targeted therapeutic approaches and expanded clinical options for treating psoriasis.

**Scheme 1:**
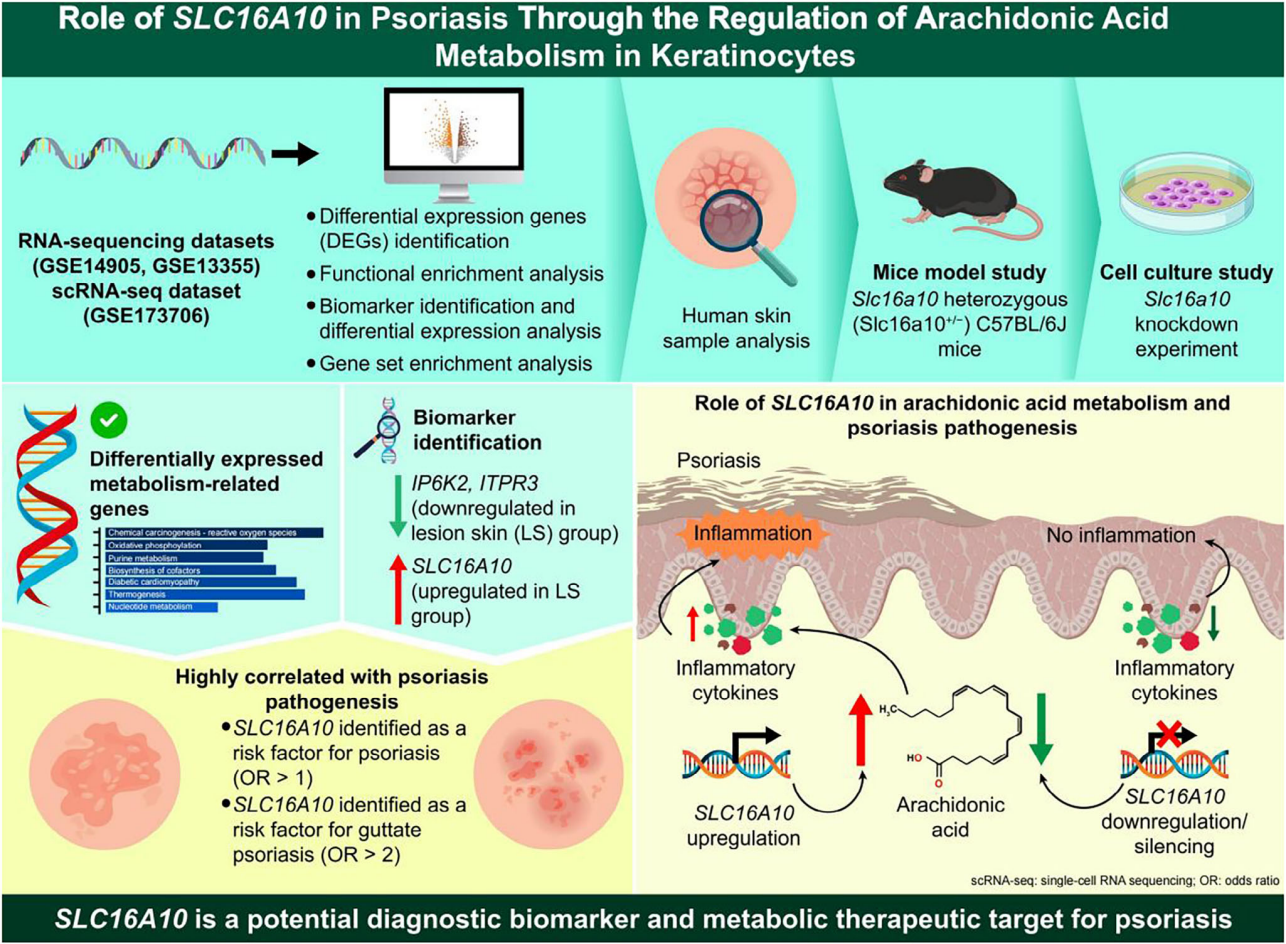
Role of SLC16A10 in psoriasis through the regulation of arachidonic acid (AA) metabolism in keratinocytes. Firstly, differentially expressed genes (DEGs) identification, functional enrichment analysis, biomarker identification, and gene set enrichment analysis were performed on RNA‐sequencing datasets (GSE14905, GSE13355, and GSE173706) to screen for differentially expressed metabolism‐related genes. Bioinformatics analysis identified key genes highly correlated with psoriasis pathogenesis. Notably, *IP6K2* and *ITPR3* were found to be downregulated in lesional skin, while *SLC16A10* was upregulated. *SLC16A10* was identified as a risk factor for psoriasis (OR > 1) and guttate psoriasis (OR > 2). Functional validation was conducted through human skin sample analysis, the exploration of *Slc16a10* heterozygous (*Slc16a10* ^+/‐^) C57BL/6J mice model, and the *Slc16a10* knockdown experiments. Mechanistic investigations revealed that *SLC16A10* upregulation promoted AA metabolism, resulting in increased production of inflammatory cytokines and subsequent psoriatic inflammation. Conversely, the downregulation or silencing of *SLC16A10* inhibited AA metabolism and alleviated inflammatory responses. The findings demonstrate that *SLC16A10* is a potential diagnostic biomarker and metabolic therapeutic target for psoriasis. Note: single‐cell RNA sequencing (scRNA‐seq); odds ratio (OR).

Therefore, in the present study, we aimed to identify differentially expressed metabolism‐related genes (DE‐MRGs) in psoriasis by extracting MRGs from public databases and performing RNA‐sequencing (RNA‐seq) analyses. We performed various bioinformatics analyses to comprehensively explore the pathogenesis of psoriasis, specifically the role of DE‐MRGs, and identify potential biomarkers for clinically targeted metabolic therapies. Additionally, we conducted comprehensive functional experiments to verify the potential roles of the identified key genes and investigated transcriptomic changes in KCs. The detailed research **Scheme** **1** is presented as follows:

## 2 Results

### 2.1 Identification of DEGs and Functional Enrichment Analysis for DE‐MRGs

**Figure 1:**
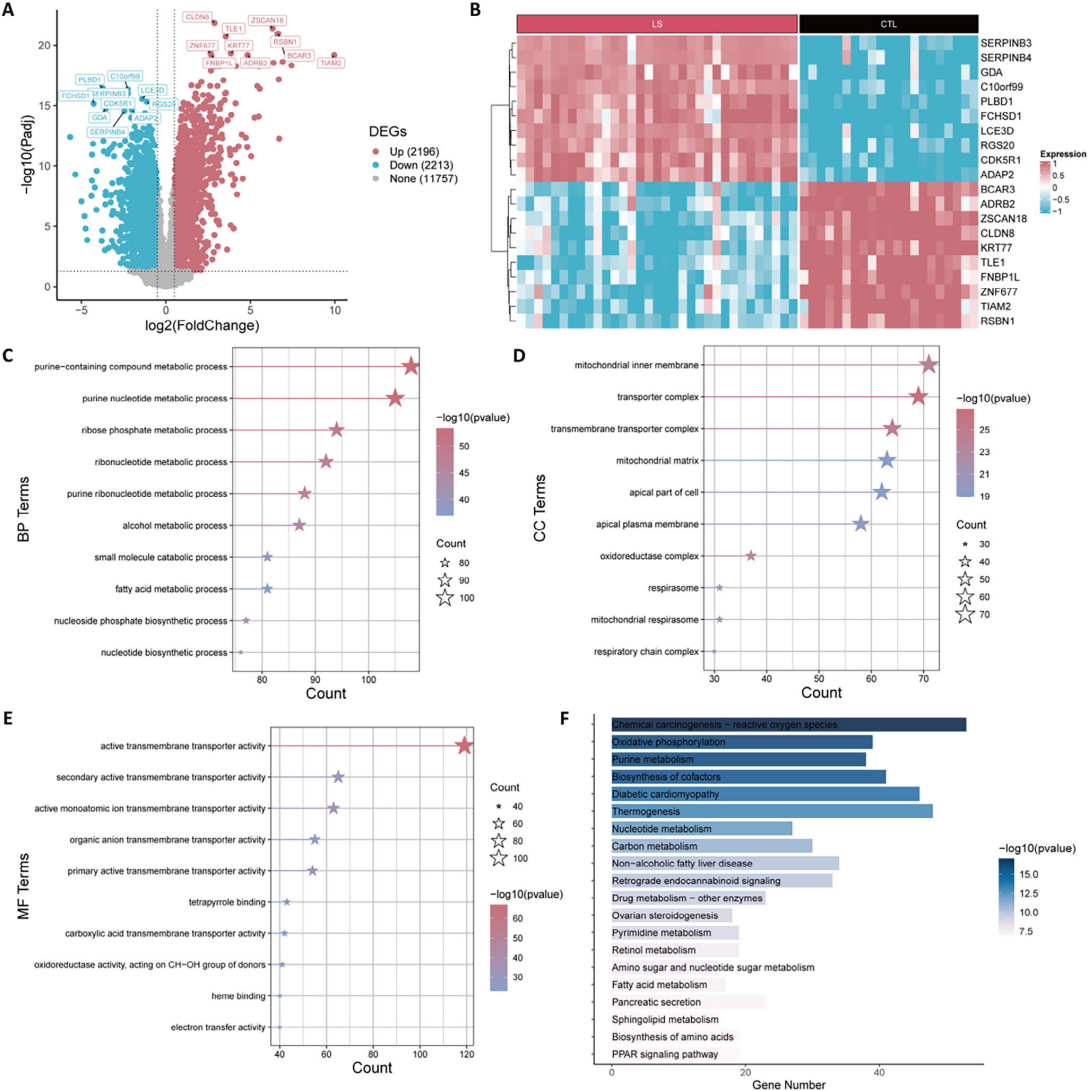
Overview of the identification and enrichment analyses of DEGs. A) Volcano plot of DEGs between the LS and CTL groups; B) Heatmap of the TOP10 upregulated and downregulated DEGs between the LS and CTL groups; C) Star plot showing the TOP10 GO‐BP terms related to DE‐MRGs; D) Star plot of the TOP10 GO‐CC terms related to DE‐MRGs; E) Star plot of the TOP10 GO‐MF terms related to DE‐MRGs; F) Bar chart of the TOP20 KEGG enriched pathways related to DE‐MRGs. Note: The horizontal coordinates of (B) show sample grouping, where the colors pink and black represent LS and CTL samples, respectively, and the vertical coordinates are DE‐MRGs. DEG: differentially expressed genes; LS, lesion skin; CTL, control (healthy skin); DE‐MRGs, differentially expressed metabolism‐related genes; GO, Gene Ontology; KEGG, Kyoto Encyclopedia of Genes and Genome; BP, biological processes; MF, molecular function; CC, cellular component.

Differential expression analysis using the GSE14905 dataset revealed 4,409 DEGs, of which 2,196 DEGs were upregulated and 2,213 DEGs were downregulated in the LS group (**Figure** **1**A). The expression patterns of the TOP 10 upregulated and downregulated DEGs were depicted in **Figure** 1B. A total of 695 DE‐MRGs overlapped between DEGs and MRGs (Figure S1, Supporting Information).

GO annotation of DE‐MRGs revealed enrichment of 1,068 biological processes (BPs), 105 cellular components (CCs), and 337 molecular functions (MFs). Analysis of the TOP 10 GO enriched BP terms (Figure 1C) revealed a strong correlation between DE‐MRGs and purine‐containing compounds and purine nucleotide metabolism. In terms of CCs, DE‐MRGs were highly enriched in the mitochondrial inner membrane, transporter complex, and transmembrane transporter complex (Figure 1D). In terms of MFs, DE‐MRGs demonstrated a significant association with active transmembrane transporter activity (Figure 1E). KEGG enrichment analysis yielded 149 related pathways. Among these pathways, DE‐MRGs were strongly correlated with signaling pathways, including chemical carcinogenesis, reactive oxygen species, oxidative phosphorylation, purine metabolism, diabetic cardiomyopathy, and nucleotide metabolism (Figure 1F). Thus, those DE‐MRGs are involved in the pathways concerning psoriasis pathogenesis,^[^ ^1^, ^16^, ^17^, ^18^, ^19^ ^]^ and warrant further exploration.

### 2.2 Identification of Key Biomarkers Through MR and Evaluation of Their Diagnostic Value

**Figure 2:**
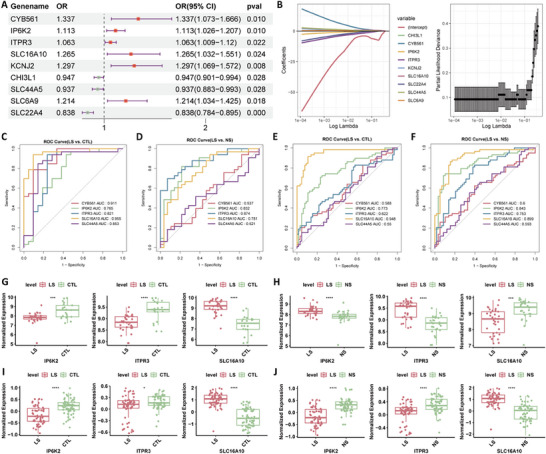
Identification of key DE‐MRGs as biomarkers through MR and evaluation of their diagnostic value. A) Forest plot of the MR analysis results for DE‐MRGs with psoriasis; B) Changes in the coefficients of individual gene variables after LASSO‐Cox regression (left) of feature genes and cross‐validation results after LASSO‐Cox regression (right) of feature genes; C) ROC curve analysis of feature genes in distinguishing between the LS and CTL groups using the GSE14905 dataset; D) ROC curve analysis of feature genes in distinguishing between the LS and NS groups using the GSE14905 dataset; E) ROC curve analysis of feature genes in distinguishing between the LS and CTL groups using the GSE13355 dataset; F) ROC curve analysis of feature genes in distinguishing LS and NS groups using the GSE13355 dataset; G) Boxplot of biomarker expression between the LS and CTL groups using the GSE14905 dataset; H) Boxplot of biomarker expression between the LS and NS groups using the GSE14905 dataset; I) Boxplot of biomarker expression between the LS and CTL groups using the GSE13355 dataset; J) Boxplot of biomarker expression between the LS and NS groups using the GSE13355 dataset. ^*^ *P* < 0.05, ^**^ *P* < 0.01, ^***^ *P* < 0.001, ^****^ *P* < 0.0001. MR, Mendelian randomization; DE‐MRGs, differentially expressed metabolism‐related genes; ROC, receiver operating characteristic; LS, lesion skin; CTL, control (healthy skin).

MR analysis revealed a significant causal association of 9 out of 695 DE‐MRGs with psoriasis (*P* < 0.05; **Figure** **2**A). Specifically, cytochrome B561 (*CYB561)*, Potassium Inwardly Rectifying Channel Subfamily J Member 2 (*KCNJ2*), Solute Carrier Family 16 Member 10 (*SLC16A10*), Solute Carrier Family 6 Member 9 (*SLC6A9*), Inositol Hexakisphosphate Kinase 2 (*IP6K2*), and Inositol 1,4,5‐Trisphosphate Receptor Type 3 (*ITPR3*) were identified as risk factors for psoriasis (odds ratio [OR] > 1), as the upregulated expression of these genes increased the risk of psoriasis. In contrast, chitinase 3‐like 1 (*CHI3L1*), Solute Carrier Family 44 Member 5 (*SLC44A5*), and Solute Carrier Family 22 Member 4 (*SLC22A4*) emerged as protective factors for psoriasis (OR < 1). Moreover, scatter plots from the MR analysis indicated consistent slope direction across all exposures. The intercepts of the IVW were all close to zero, indicating minimal horizontal pleiotropy and supporting the reliability of the MR results (Figure S2, Supporting Information). Horizontal pleiotropy analyses further confirmed the absence of horizontal pleiotropy (*P* > 0.05); however, heterogeneity analysis indicated that there was heterogeneity in some DE‐MRGs, as indicated by Cochran's Q test (Q_ p < 0.05) (Table S3, Supporting Information). Leave‐one‐out analysis indicated the absence of severely biased SNPs (Figure S3, Supporting Information).

LASSO logistic regression of the nine significant exposure variables yielded five feature genes: *CYB561*, *IP6K2*, *ITPR3*, *SLC16A10*, and *SLC44A5* (Figure 2B). Subsequently, we evaluated the diagnostic value of these feature genes using the GSE14905 set. The area under the ROC curves (AUC) was greater than 0.7, indicating that all five feature genes effectively differentiated between the LS and CTL groups, demonstrating a strong diagnostic value (Figure 2C, D). Additionally, *SLC16A10*, *IP6K2*, and *ITPR3* effectively distinguished between the LS and NS groups, indicating their superior diagnostic value compared to *CYB561* and *SLC44A5*. We subsequently validated the diagnostic value of these feature genes using the GSE13355 dataset. Notably, *SLC16A10*, *IP6K2*, and *ITPR3* retained their diagnostic value (AUC > 0.6) in distinguishing between the LS and NS and between the LS and CTL groups (Figure 2E,F). Therefore, we selected *SLC16A10*, *IP6K2*, and *ITPR3* as key biomarkers with high potential for subsequent analyses.

### 2.3 Differential Expression Analyses and MR Validation for Key Biomarkers

Differential expression analysis using the GSE14905 dataset revealed downregulation of *IP6K2* and *ITPR3* expression in the LS group compared with that in the CTL group, whereas *SLC16A10* expression was upregulated (Figure 2G). The opposite trend was observed between the LS and NS groups (Figure 2H). Validation using the GSE13355 dataset indicated that the trend of biomarker expression between the LS and CTL groups remained consistent with that observed in the GSE14905 dataset (Figure 2I), whereas an opposite trend was observed between the LS and NS groups (Figure 2J). Notably, the expression of *SLC16A10* in the LS and CTL groups was consistent with that observed in the MR analysis. Thus, we mainly focused on exploring the potential functions of *SLC16A10* in the LS and CTL groups.

We performed an additional MR analysis to validate the causal relationship between the biomarkers and psoriasis, focusing on outcomes such as psoriasis vulgaris and psoriasis subtypes, owing to limitations in dataset collection. The analysis demonstrated a significant causal relationship between *IP6K2*, *ITPR3*, and psoriasis vulgaris (*P* < 0.05). *ITPR3* expression was also causally associated with psoriatic arthritis (Table S1, Supporting Information). All three biomarkers were identified as risk factors in the MR validation analysis (OR > 1). Moreover, *SLC16A10* expression was associated with a significant risk of guttate psoriasis (OR > 2). Sensitivity analyses did not reveal any factors affecting the reliability of the results (Table S4, Figure S4, Supporting Information).

### 2.4 GSEA for Key Biomarkers

**Figure 3:**
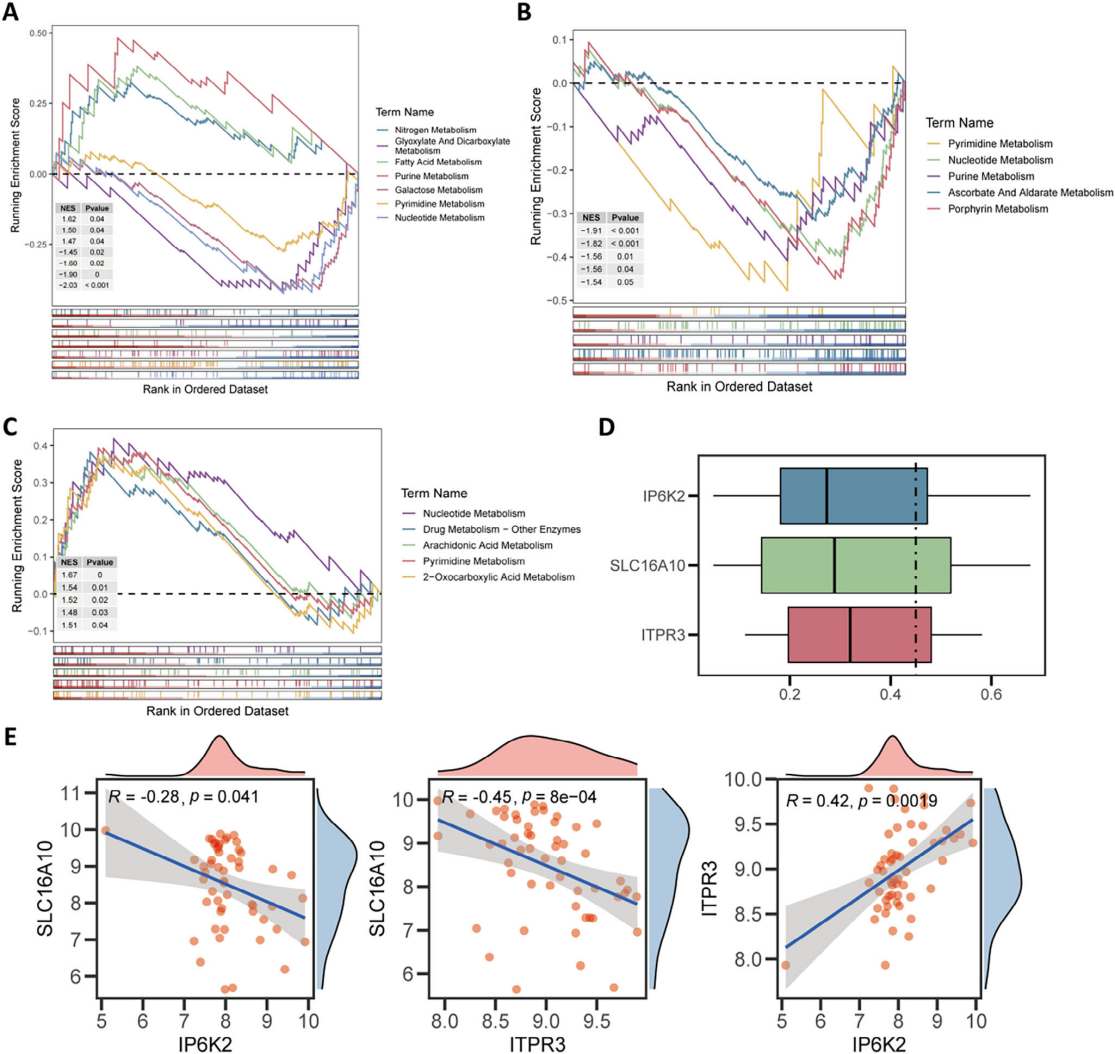
GSEA, functional similarity, and Spearman's correlation analyses for the identified biomarkers. A) Enrichplot of *IP6K2* based on KEGG enrichment; B) Enrichplot of *ITPR3* based on KEGG enrichment; C) Enrichplot of *SLC16A10* based on KEGG enrichment; D) Bar chart showing functional similarity among biomarkers; E) Scatter plots showing correlation among biomarkers. GSEA, gene set enrichment analysis; KEGG, Kyoto Encyclopedia of Genes and Genomes.

We performed GSEA for the identified biomarkers to explore the related pathways and functions. The results indicated that *IP6K2* expression was significantly correlated with nitrogen, glyoxylate, dicarboxylic acid, fatty acid, purine, galactose, pyrimidine, and nucleotide metabolism (**Figure** **3**A). *ITPR3* expression exhibited significant correlations with pyrimidine, nucleotide, and purine metabolism (Figure 3B). Moreover, *SLC16A10* expression was significantly enriched in pathways related to nucleotide, AAs, pyrimidine, and 2‐oxalate metabolism (Figure 3C). The functional similarity between the three biomarkers exceeded 0.4 (Figure 3D). Combined with the results of the enrichment analysis, the similar functions of the biomarkers appear to be focused on pyrimidine, nucleotide, and energy metabolism pathways. Spearman's correlation analysis revealed significant negative correlations between *SLC16A10* and both *IP6K2* and *ITPR3*, whereas *IP6K2* and *ITPR3* exhibited significant positive correlations. These results are consistent with the trends observed in biomarker expression between the LS and CTL groups (Figure 3E). Based on the expression of *IP6K2* and *ITPR3* and correlation analyses, we focused on the functional exploration of the key biomarker *SLC16A10* in subsequent analyses.

### 2.5 Functional Exploration of Key Cells with Differential Expression of SLC16A10 in scRNA‐Seq

**Figure 4:**
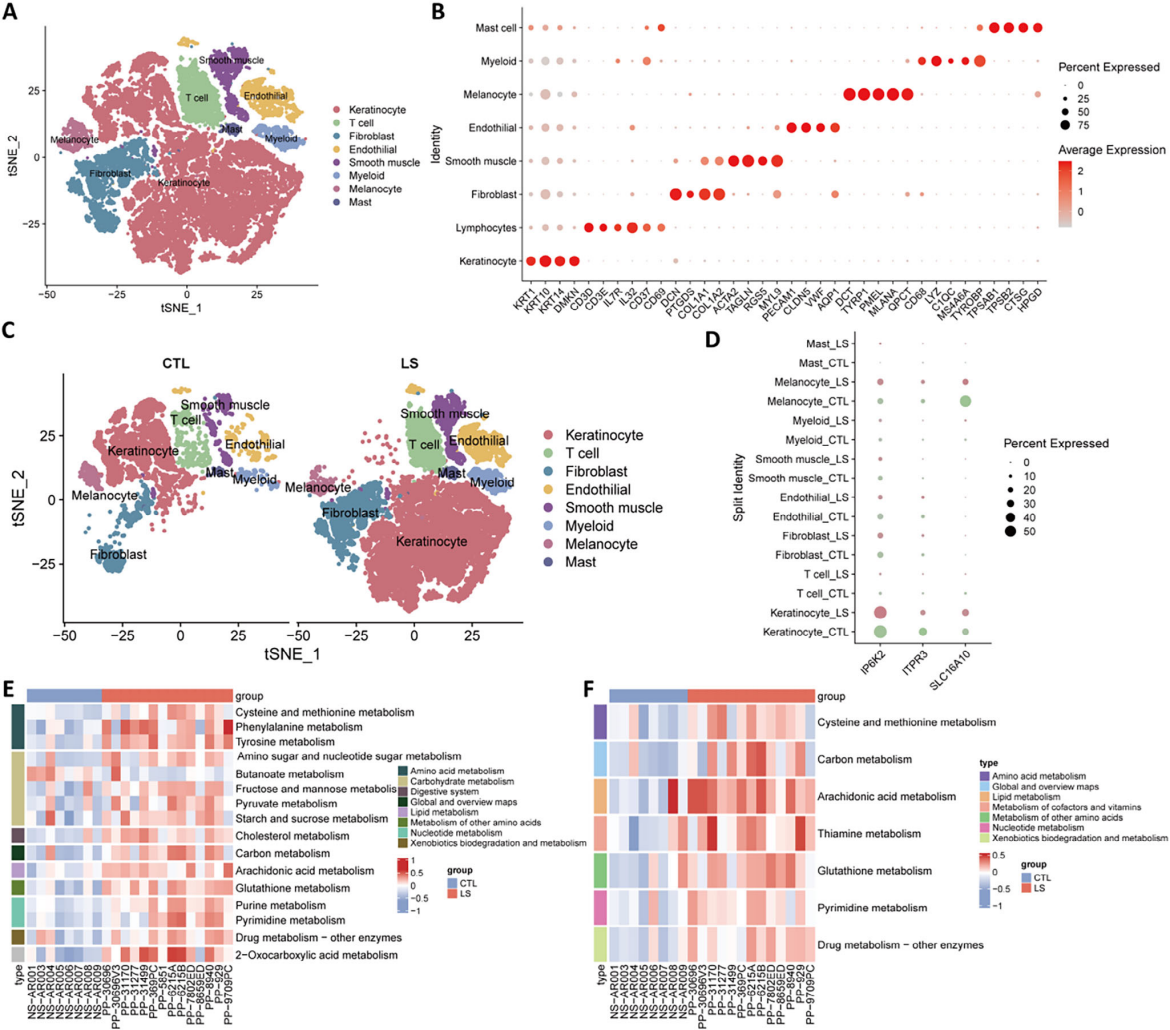
Overview of single‐cell RNA‐sequencing analyses in psoriasis datasets. A) t‐SNE of eight cell subpopulations in the GSE173706 dataset; B) Dot plot of marker gene expression in each cell subpopulation; C) t‐SNE of eight cell subpopulations between the LS and CTL groups in the GSE173706 dataset; D) Dot plot of the expression of the three identified biomarkers in cell subpopulations between the LS and CTL groups; E) Heatmap of metabolic pathway differences in keratinocytes between the LS and CTL groups; F) Heatmap of metabolic pathway differences in melanocytes between the LS and CTL groups. t‐SNE: t‐distributed Stochastic Neighbor Embedding; LS, lesion skin; CTL, control (healthy skin).

We clustered cells using unsupervised clustering at a default resolution of 1, resulting in 27 clusters (Figure S5A, Supporting Information). After cell annotation, we identified 27 clusters corresponding to 8 cell subpopulations: KC, lymphocytes, fibroblasts, smooth muscle, endothelial cells, MC, myeloid cells, and mast cells (**Figure** **4**A,B). KCs exhibited a distinct separation in distribution between the LS and CTL groups, indicating that KCs in psoriasis undergo fundamental transcriptome changes (Figure 4C). The remaining cell subpopulations overlapped between both groups, with a relatively high degree of MC separation (Figure 4C). Notably, all three biomarkers were expressed in KCs, with *IP6K2* exhibiting a significantly higher expression than the other two biomarkers (Figure 4D). *SLC16A10* expression was significantly higher than that of *IP6K2* and *ITPR3* in MCs. Moreover, differential scRNA‐seq analyses revealed that *SLC16A10* expression was significantly upregulated in KCs, whereas it was significantly downregulated in MCs (Figure S5B,C, Supporting Information). Psoriasis is characterized by significant transcriptomic changes in KCs, however, the impact of MC transcriptomic changes in psoriasis remains understudied.^[^ ^20^ ^]^ Therefore, we analyzed KCs and MCs as key cell types.

GSVA of metabolic pathways in KCs of the LS group revealed significant enrichment in 13 metabolic pathways (Figure 4E). Among them, the phenylalanine, tyrosine, AA, glutathione, and 2‐oxalate metabolic pathways were more significantly enriched than the other metabolic pathways. Seven metabolic pathways were significantly enriched in the MCs of the LS group, with AA metabolism exhibiting the most significant enrichment (Figure 4F). Notably, the metabolic pathways enriched in both KCs and MCs were related to psoriasis pathogenesis, and these pathways were highly consistent with those observed for the three identified biomarkers.

### 2.6 Pseudo‐Time Analyses Demonstrate Differentiation Trajectories of KCs and MCs in Psoriasis Pathogenesis

**Figure 5:**
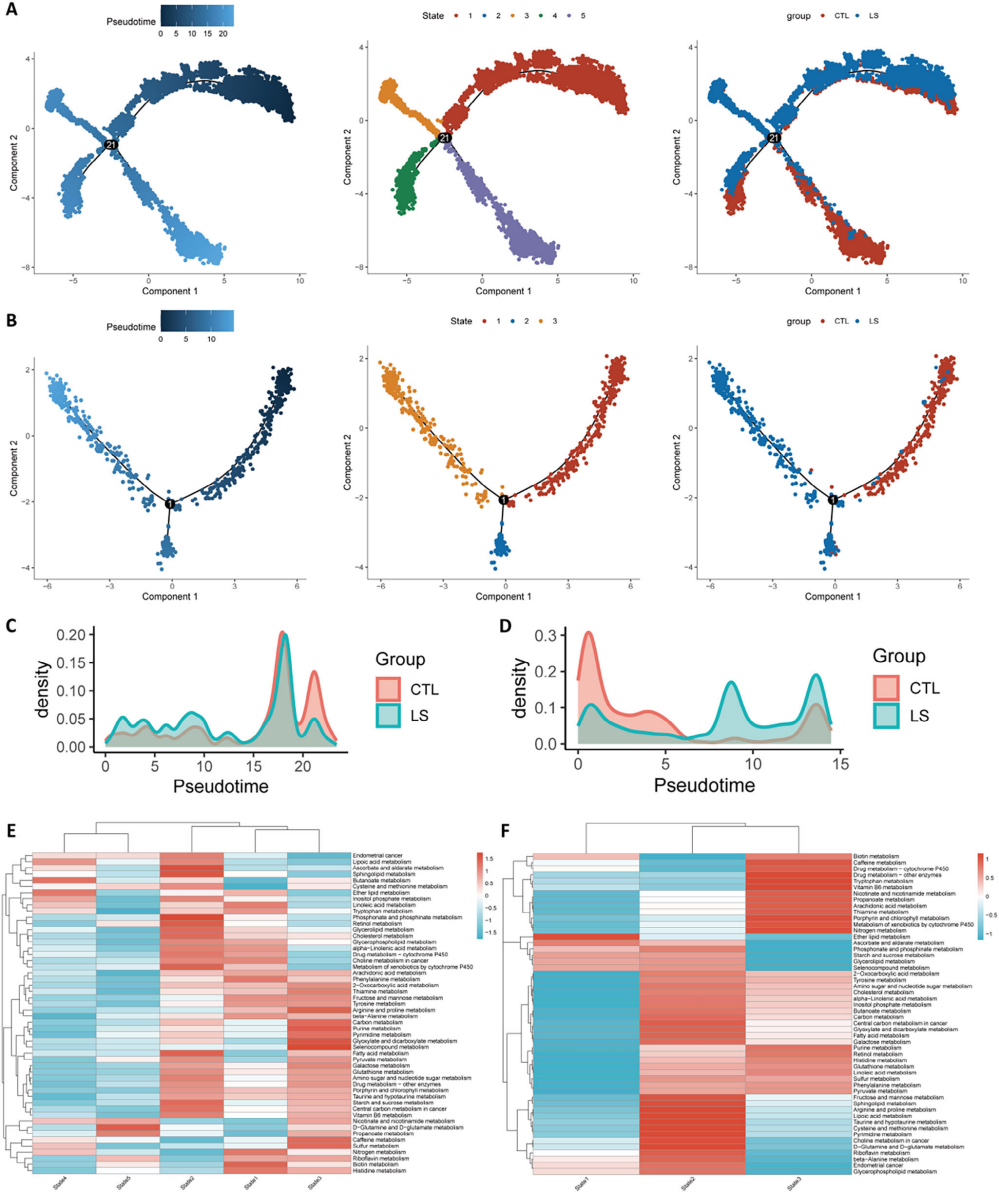
Overview of pseudo‐time analysis. A) Pseudo‐time analysis for KCs; B) Pseudo‐time analysis for MCs. C) Cell density curve for KCs based on the pseudo‐time analysis; D) Cell density curve for MCs based on the pseudo‐time analysis; E) Heatmap of the GSVA results for KCs based on the pseudo‐time analysis; F) Heatmap of the GSVA results for MCs based on the pseudo‐time analysis. Note: The lighter blue color of the pseudo‐time analysis Left) in (A) and (B) represents a higher degree of differentiation. KCs, keratinocytes; MCs, melanocytes; GSVA, gene set variation analysis.

We performed a pseudo‐time analysis to investigate the differentiation trajectories of KCs and MCs in the context of psoriasis. This analysis revealed that KCs followed a differentiation trend from right to left and top to bottom, with state five representing the highest degree of differentiation (**Figure** **5**A). KCs were distributed across various stages of differentiation; however, the proportion of cells with the highest degree of differentiation was significantly lower in the disease group than in the CTL group. This suggests that KCs in the LS samples did not progress to the appropriate functional state (Figure 5A–C), indicating a disruption in the differentiation process associated with psoriasis. These results corroborated previous findings indicating that KCs undergo fundamental transcriptomic changes during psoriasis. Moreover, our GSVA results demonstrated that functional pathways, involving lipid, energy, and amino acid metabolism, in KCs at an early stage of differentiation (states 1–3) were more active than those at a later stage of differentiation (Figure 5E). Notably, the difference in the degree of active AA metabolism was consistent with our previous analysis (Figure 5E). Combined with the cell density distribution, our results indicate that KCs in the LS group were more active and engaged in functional pathways corresponding to the GSEA results for *SLC16A10*. Therefore, we speculated that the significant upregulation of *SLC16A10* in KCs might promote psoriasis development by affecting intracellular metabolic homeostasis.

The developmental trajectories of MCs exhibited a right‐to‐left differentiation trend, with the highest degree of differentiation in state three. MCs in the LS group demonstrated more pronounced differentiation compared to those in the CTL group, with the differentiation trajectories of the two groups diverging, suggesting the presence of two distinct differentiation states (Figure 5B–D). Moreover, the GSVA results of MCs revealed that the activity of AA metabolism was generally elevated in the LS group compared to that in the CTL group, with a notable increase in state three (Figure 5F). However, AA metabolism in state two was relatively inactive, suggesting the complex conditions in MCs during psoriasis development.

### 2.7 Validation of the Upregulation of SLC16A10 Expression and AA Content in the Epidermis of Individuals with Psoriasis

**Figure 6:**
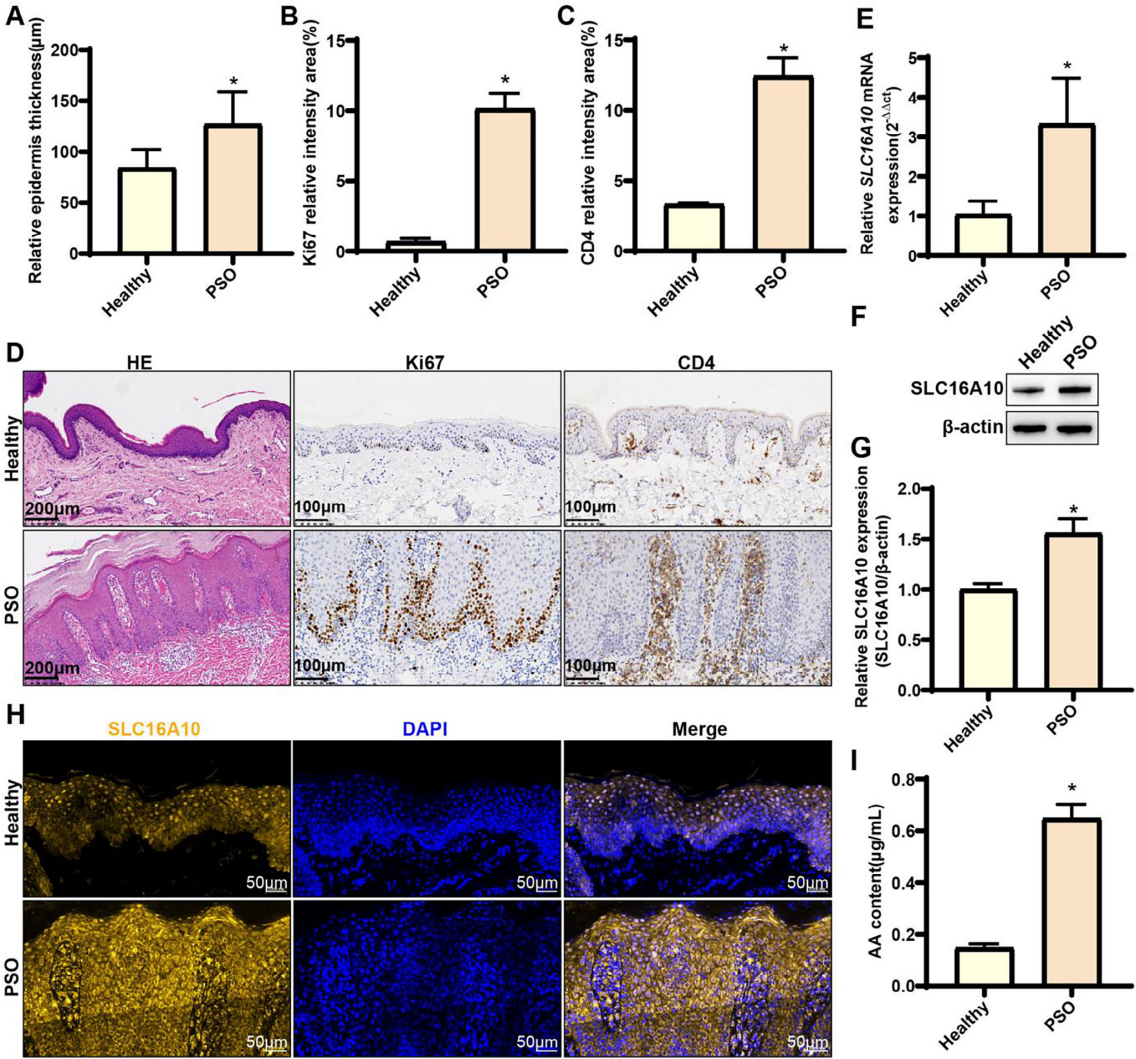
Validation of the upregulation of *SLC16A10* expression and AA content in the epidermis of individuals with psoriasis. A) Epidermal thickness, n = 6; B) Signal intensity in Ki67‐positive cells, n = 6; C) Signal intensity in CD4‐positive cells, n = 6; D) Hematoxylin and eosin staining and immunohistochemistry for Ki67 and CD4 expression; E) Reverse transcription real‐time quantitative polymerase chain reaction analysis of *SLC16A10* expression, n = 3; F) Western blotting analysis of SLC16A10 expression; G) Quantification of the western blotting results for SLC16A10, n = 3; H) Immunofluorescence staining for SLC16A10; I) Quantification of AA content, n = 3. Note: β‐actin served as the internal reference in western blotting. All experiments were conducted using epidermal tissues of healthy participants and individuals with psoriasis (PSO). AA, arachidonic acid. ^*^ *P* > 0.05, compared with healthy group.

We compared the epidermal thickness between the epidermis of individuals with psoriasis and that of healthy individuals using HE staining, which revealed a significant increase in epidermal thickness in individuals with psoriasis (**Figure** **6**A and **6**D). The relative proportional intensities of both Ki67 and CD4‐positive cells in the psoriatic epidermis were remarkably high. The tissue sections exhibited consistent results, demonstrating increased proliferation of epidermal cells in individuals with psoriasis, along with heightened inflammatory and immune responses (Figure 6B–D). Notably, both RT‐qPCR and WB analyses confirmed the distinct upregulation of SLC16A10 in the psoriatic epidermis at the gene and protein levels, respectively, in line with our bioinformatic analysis results (Figure 6E–G). Immunofluorescence results revealed significant upregulation of SLC16A10 in the psoriatic epidermis (Figure 6H; Figure S6, Supporting Information). ELISA demonstrated significantly increased AA content in the epidermis of individuals with psoriasis compared to that in healthy individuals (Figure 6I).

### 2.8 *Slc16a10* Knockdown in Psoriatic Mice Significantly Alleviates Psoriasis Severity

**Figure 7:**
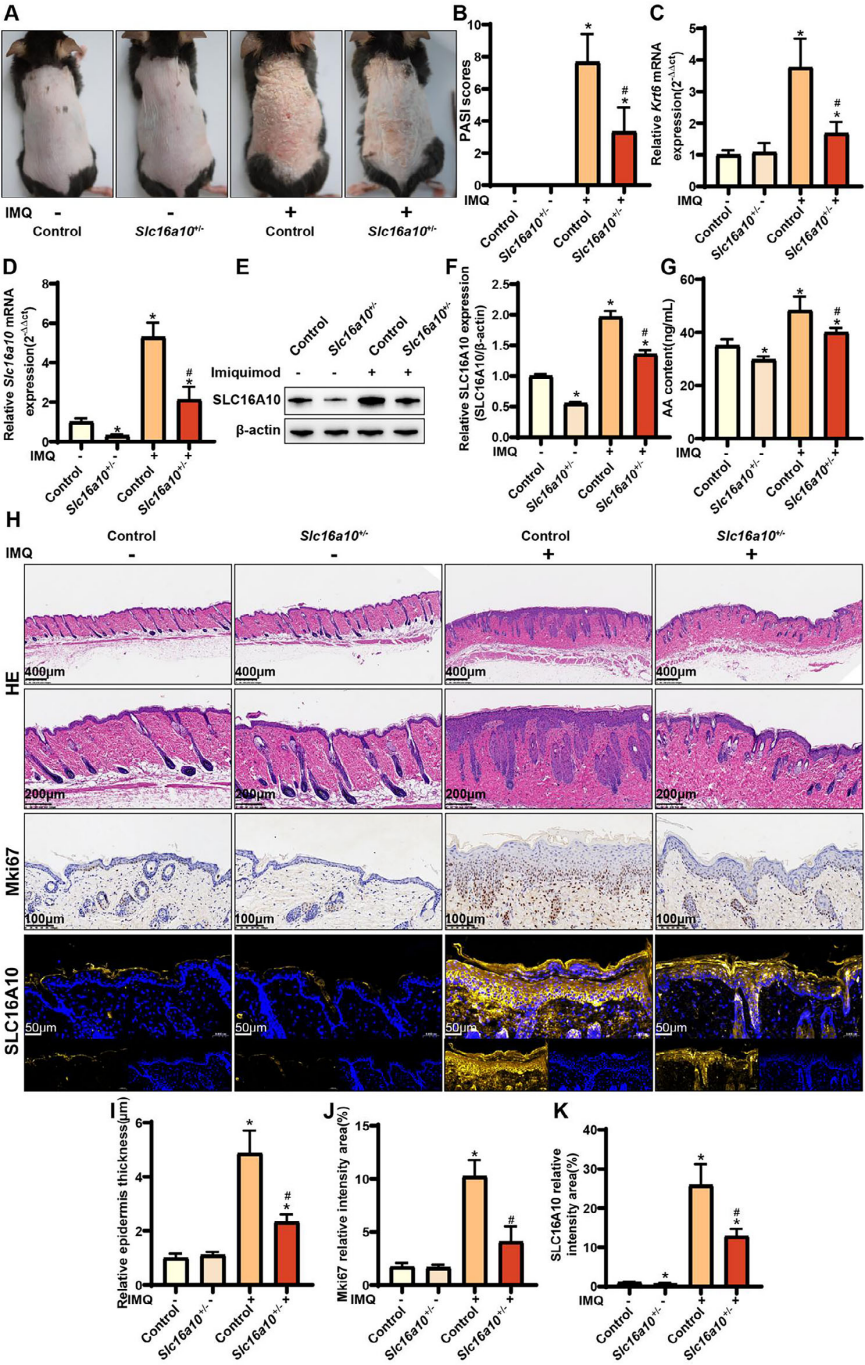
Effects of *Slc16a10* knockdown on psoriasis severity in vivo. A) Comparison of pathological manifestations in mouse models of psoriasis established via the topical application of IMQ; B) Comparison of psoriasis severity index scores, n = 6; C) RT‐qPCR analysis of *Krt6* expression, n = 6; D) RT‐qPCR analysis of *Slc16a10* expression, n = 6; E) Western blotting analysis of SLC16A10 expression; F) Quantification of the western blotting results, n = 4; G) Quantification of the AA content, n = 6; H) Hematoxylin and eosin and immunohistochemistry staining for Ki67, and immunofluorescence staining for SLC16A10; I) Epidermal thickness, n = 6; J) Signal intensity in Ki67‐positive cells, n = 6; K) Signal intensity in SLC16A10‐positive cell. Note: β‐actin served as the internal reference for western blotting. All experiments were conducted in epidermal tissues of mice under four conditions: IMQ‐, IMQ‐ *Slc16a10* ^+/−^, IMQ+, and IMQ+ *Slc16a10* ^+/−^. RT‐qPCR, real‐time quantitative polymerase chain reaction; AA, arachidonic acid. ^*^ *P* > 0.05, compared with IMQ‐ group; ^#^ *P* > 0.05, compared with IMQ+ group.

We constructed a mouse model of psoriasis‐like skin inflammation using IMQ to validate the role of Slc16a10 expression in vivo. The PSI scores of the mice increased significantly after applying IMQ. Moreover, mice were classified into four groups: IMQ‐, IMQ‐ *Slc16a10* ^+/−^, IMQ+, and IMQ+ *Slc16a10* ^+/−^. RT‐qPCR and WB results confirmed reduced *Slc16a10* gene and protein expression, respectively, following knockdown, indicating successful model construction (**Figure** **7**A,B, D–F). *K* *r* *t* *6*, a key early marker in psoriasis, promotes the hyperproliferation of KCs, enhances the inflammatory response, and activates the innate immune.^[^ ^21^ ^]^ Our results indicated that *Slc16a10* knockdown reduced the psoriasis severity in mice treated with IMQ, as evidenced by significantly reduced PSI scores and *Krt6* expression levels (Figure 7B,C). Furthermore, the AA content was significantly reduced in both *Slc16a10* knockout mouse groups (regardless of IMQ treatment) compared to that in healthy mice with psoriasis that did not undergo *Slc16a10* knockout (Figure 7G). Compared with IMQ‐mice, IMQ‐ *Slc16a10* ^+/−^ mice exhibited insignificant changes in epidermal thickness and cell proliferation, whereas *Slc16a10* expression was significantly reduced (Figure 7H–K). In contrast with IMQ+ mice, IMQ+ *Slc16a10* ^+/–^ mice exhibited a significant decrease in epidermal thickness, cell proliferation, and *Slc16a10* expression, accompanied by inhibition in cell proliferation (Figure 7H–K).

**Figure 8:**
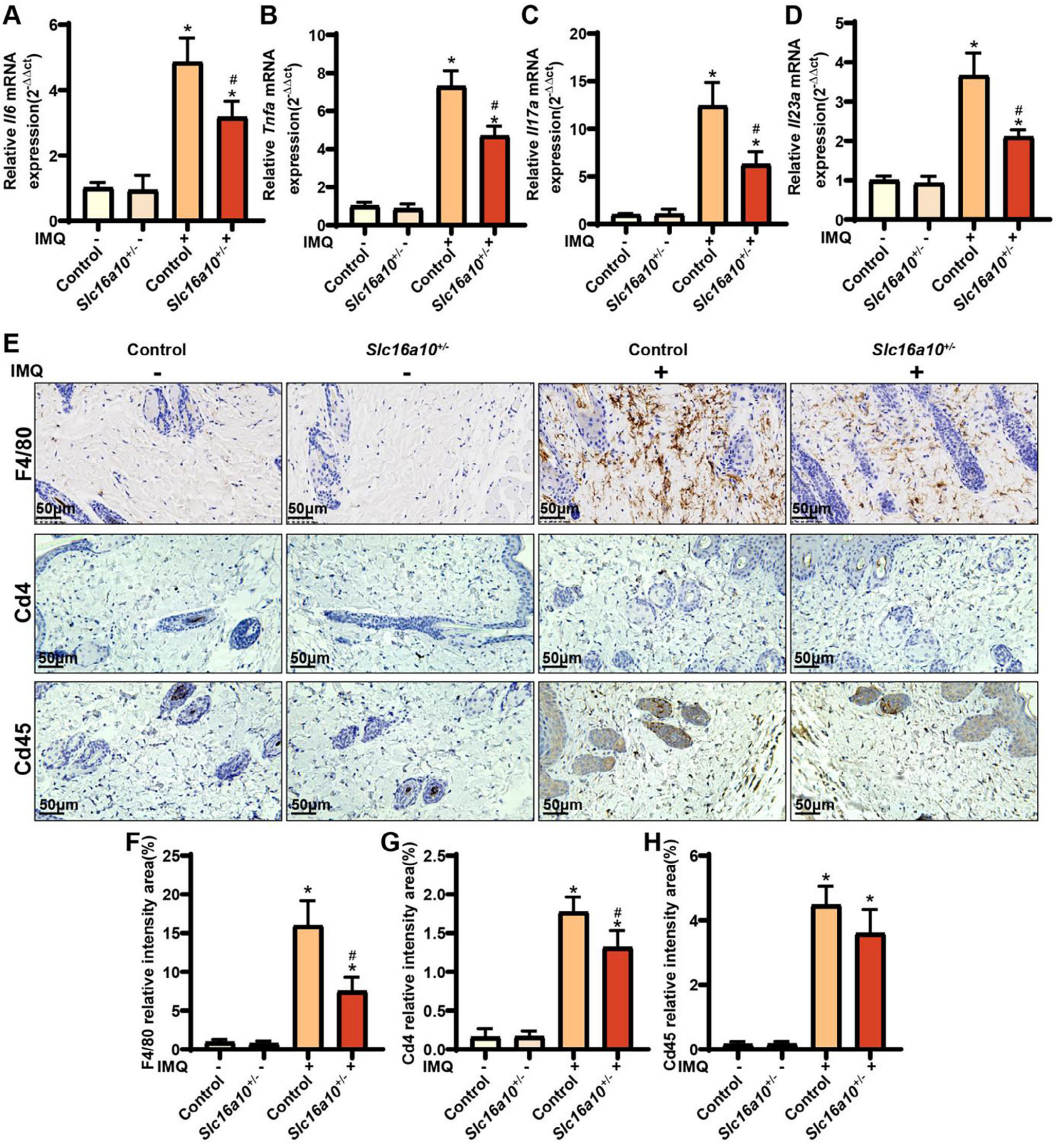
Effects of *Slc16a10* knockdown on the expression of key inflammatory indicators in vivo. A–D) Reverse transcription real‐time quantitative polymerase chain reaction analysis of *IL, Tnfa*, *Il17a*, and *Il23a* expression, n = 6; E) Immunohistochemical staining for F4/80, Cd4, and Cd45; F) Signal intensity in F4/80‐positive cells, n = 6; G) Signal intensity in CD4‐positive cells, n = 6; H) Signal intensity in CD45‐positive cells, n = 6. Note: All experiments were conducted in epidermal tissues of mice under four conditions: IMQ‐, IMQ‐ *Slc16a10* ^+/−^, IMQ+, and IMQ+ *Slc16a10* ^+/−^. ^*^ *P* > 0.05, compared with IMQ‐ group; ^#^ *P* > 0.05, compared with IMQ+ group.

We also analyzed the gene expression of the key inflammatory indicators *Il6, Tnfa, Il17a*, *and Il23a*, to assess changes in inflammatory infiltration in mice. The expression levels of *Il6*, *Tnfa*, *Il17a*, and *Il23a* were significantly elevated in IMQ+ mice compared with those in IMQ+*Slc16a10* ^+/−^ mice (**Figure** **8**A–D). Immunohistochemistry staining revealed significant downregulation in the expression of F4/80, Cd4, and Cd45 in IMQ+ *Slc16a10* ^+/−^ mice (Figure 8E–H). These results indicated that *Slc16a10* knockdown significantly reduced psoriasis severity and inhibited excessive inflammatory and immune responses, suggesting the potential of SLC16A10 as a therapeutic target for psoriasis.

### 2.9 *SLC16A10* Silencing Reduces Hyperinflammation in KCs and Melanogenesis in MCs

**Figure 9:**
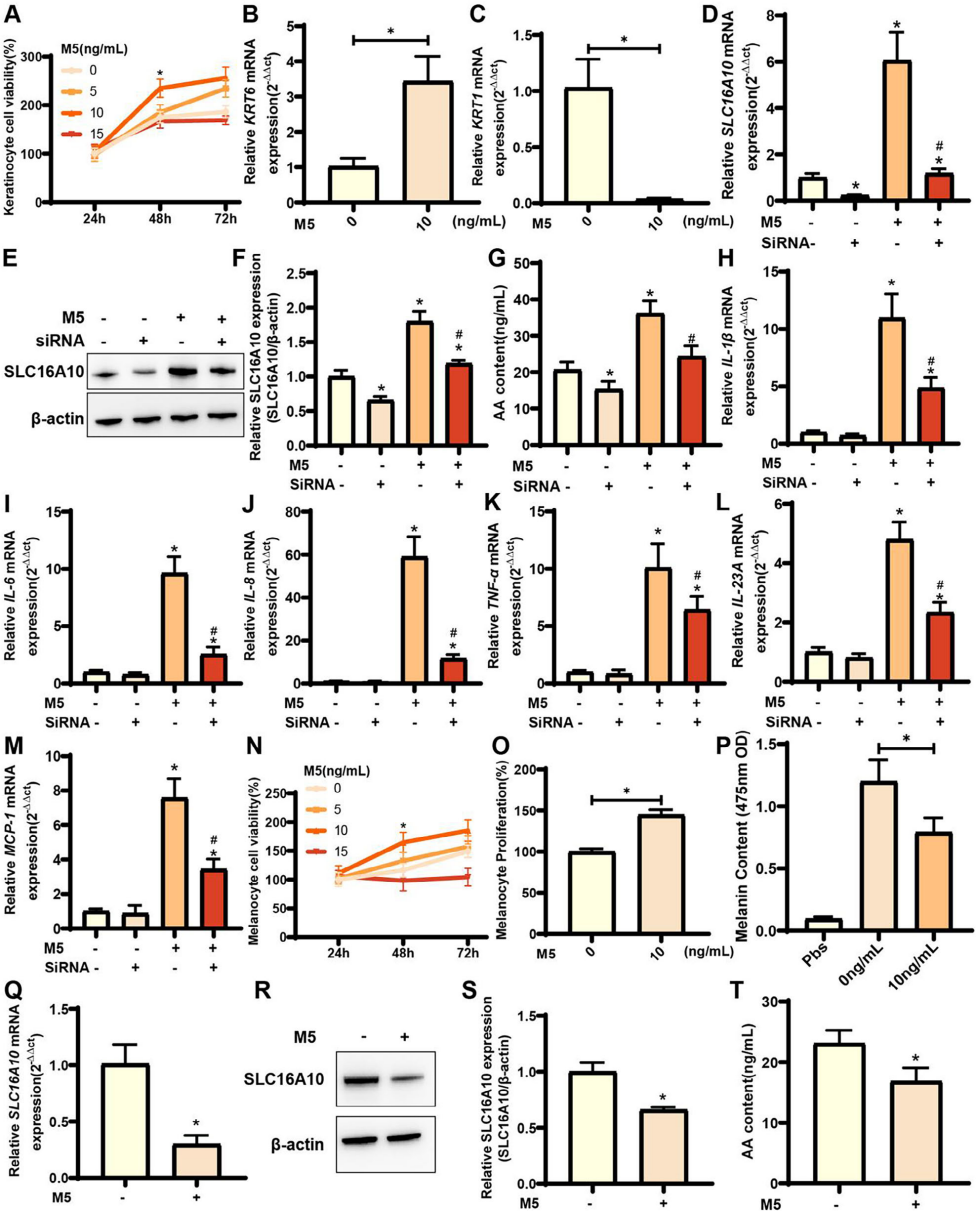
Effects of *SLC16A10* silencing on hyperinflammation in KCs and melanogenesis in MCs. A) Viability of KCs exposed to different concentrations and duration of M5, n = 6; B) RT‐qPCR analysis for *KRT6* in KCs under 10 ng mL^−1^ M5 concentrations, n = 6; C) RT‐qPCR analysis of *KRT1* expression in KCs under 10 ng mL^∐1^ M5 concentrations, n = 6; D) RT‐qPCR analysis of *SLC16A10* expression in KCs under different conditions, n = 6; E) Western blotting analysis of SLC16A10 expression in KCs under different conditions; F) Quantification of western blotting results for SLC16A10 in KCs under different conditions (M5‐/M5+ and/or siSLC16A10), n = 4; G) Quantification of AA content in KCs under different conditions, n = 6; H–M) RT‐qPCR analysis of *IL‐1β*, *IL‐6*, *IL‐8, TNF‐α*, *IL‐23A* and *MCP‐1* expression in KCs under different conditions (M5‐/M5+ and/or siSLC16A10), n = 6; N) Viability of MCs under different exposure time and M5 concentrations, n = 6; O) Quantification of MC proliferation under 10 ng mL^−1^ M5 concentrations, n = 6; P) Quantification of melanin content in MCs under 10 ng mL^−1^ M5 concentrations, n = 6; Q) RT‐qPCR analysis for *SLC16A10* expression in MCs under 10 ng mL^−1^ M5 concentrations, n = 6; R) Western blotting analysis of SLC16A10 expression in MCs under 10 ng mL^−1^ M5 concentrations; S) Quantification of the western blotting results for SLC16A10 in MCs under 10 ng mL^−1^ M5 concentrations, n = 3; T) Quantification of the AA content in MCs under different M5 concentrations, n = 6. Note: β‐actin was used as the internal reference in western blotting. RT‐qPCR, real‐time quantitative polymerase chain reaction; MCs, melanocytes; KCs, keratinocytes; AA, arachidonic acid. ^*^ *P* > 0.05, compared with M5‐ group; ^#^ *P* > 0.05, compared with M5+ group.

We stimulated KCs using M5, a cytokine cocktail, to mimic the inflammatory psoriatic environment. We observed the most significant increase in KC viability at 48 h at an M5 concentration of 10 ng mL^−1^ (**Figure** **9**A). Therefore, we stimulated the cells under these conditions for the keratin expression assay with *SLC16A10* silencing. KRT1 is a keratin protein in the healthy epidermis and acts as a negative regulator of inflammation.^[^ ^22^ ^]^ *KRT6* expression was significantly upregulated in the M5‐stimulated group compared with that in the control group, whereas *KRT1* expression exhibited the opposite trend (Figure 9B,C). We also determined SLC16A10 expression after silencing it under each condition using RT‐qPCR and WB. The expression of SLC16A10 was significantly decreased in the M5‐ siSLC16A10 group compared with that in the M5‐ group, whereas it was significantly increased in the M5+ group (Figure 9D–F). Notably, the expression of SLC16A10 in the M5+ siSLC16A10 group was markedly downregulated compared to that in the M5+ group (Figure 9D–F). Moreover, the AA content was significantly reduced after *SLC16A10* silencing compared to that in the M5+ and M5‐ groups (Figure 9G). The expressions of inflammatory indicators (IL‐1β, IL‐6, IL‐8, TNF‐α, IL23A, and MCP‐1) in the M5+ siSLC16A10 group were also significantly decreased (Figure 9H–M). These results suggested that *SLC16A10* silencing could reduce AA content and the intensity of inflammatory responses in KCs under inflammatory stimulation.

We also stimulated MCs using M5 and observed the highest MC viability at 48 h and 10 ng mL^−1^ (Figure 9N). Therefore, we used these conditions in subsequent experiments. Compared to the M5‐ group, we observed a significant increase in MC proliferation and a significant decrease in melanogenesis in the M5+ groups (Figure 9O,P). RT‐qPCR and WB analyses revealed a significant decrease in SLC16A10 expression following M5 stimulation and a significant decrease in AA content, consistent with the trend observed for SLC16A10 expression (Figure 9Q–T).

### 2.10 The Role of SLC16A10 in Modulating T_3_ Uptake and Inflammatory Responses in HaCaT Cells

**Figure 10:**
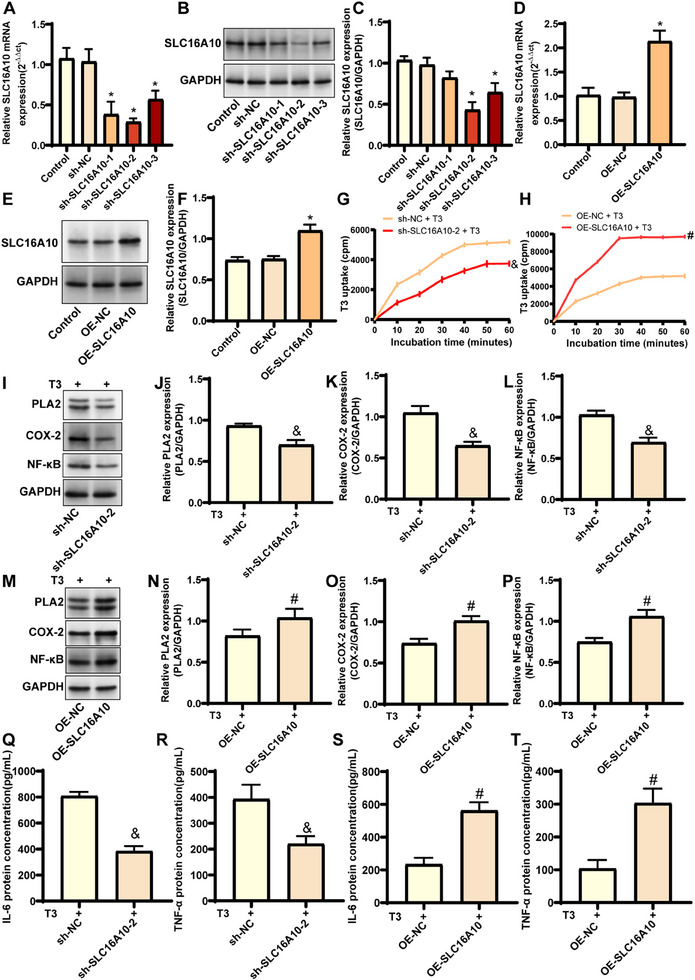
SLC16A10 modulates T_3_ uptake and inflammatory responses in HaCaT cells. A) RT‐qPCR analysis of *SLC16A10* mRNA expression in knockdown cells, n = 4; B) Representative western blot showing SLC16A10 protein levels in HaCaT cells transfected with sh‐NC, sh‐SLC16A10‐1, sh‐SLC16A10‐2, or sh‐SLC16A10‐3. GAPDH served as loading control; C) Quantification of SLC16A10 protein expression relative to GAPDH in knockdown cells, n = 3; D) RT‐qPCR analysis of *SLC16A10* mRNA expression in overexpression cells, n = 4; E) Representative western blot showing SLC16A10 protein levels in OE‐NC and OE‐SLC16A10 cells; F) Quantification of SLC16A10 protein expression in overexpression cells, n = 4; G) Time‐course analysis of ^125^I‐T_3_ uptake in sh‐NC and sh‐SLC16A10‐2 cells, n = 3; H) Time‐course analysis of ^125^I‐T_3_ uptake in OE‐NC and OE‐SLC16A10 cells. Radioactivity measured at indicated time points and expressed as counts per minute (cpm), n = 3; I) Representative western blot showing PLA2, COX‐2, and NF‐κB protein levels in T_3_‐treated sh‐NC and sh‐SLC16A10‐2 cells; J–L) Quantification of PLA2, COX‐2, and NF‐κB protein expression relative to GAPDH in knockdown cells, n = 3; M) Representative western blot showing inflammatory markers in T_3_‐treated OE‐NC and OE‐SLC16A10 cells; N–P) Quantification of PLA2, COX‐2, and NF‐κB protein expression in overexpression cells, n = 3; Q–T) ELISA analysis of IL‐6 and TNF‐α protein concentrations in culture supernatants from knockdown and overexpression cells treated with T_3_.^*^ *P* > 0.05, compared with Control group; ^&^ *P* > 0.05, compared with sh‐NC group; ^#^ *P* > 0.05, compared with OE‐NC group.

To further validate the impact of SLC16A10 on T_3_ uptake and inflammatory responses, we successfully established stable *SLC16A10* knockdown and overexpression cell lines in the HaCaT cell line using a shRNA viral infection strategy. Additionally, RT‐qPCR and western blot analyses (**Figure** **10**A–F) demonstrated that shSLC16A10‐2 exhibited the highest knockdown efficiency, which was therefore used for subsequent phenotypic experiments. To investigate the role of SLC16A10 in T_3_ uptake, we introduced exogenous T_3_ labeled with ^125^I into the stable HaCaT cell lines. By measuring the radioactivity in the cells, we found that knocking down *SLC16A10* significantly diminished the uptake of T_3_ by HaCaT cells (Figure 10G). Conversely, overexpression of *SLC16A10* markedly enhanced the uptake of T_3_ (Figure 10H). Following the addition of T_3_, we also examined the expression of inflammation‐related markers. Western blot analyses indicated that the knockdown of *SLC16A10* led to a significant decrease in the expression of PLA2, COX‐2, and NF‐κB, while the overexpression of *SLC16A10* markedly promoted their expression (Figure 10I–P). Moreover, ELISA results indicated that the levels of IL‐6 and TNF‐α decreased with reduced expression of *SLC16A10*, while they increased as *SLC16A10* expression was elevated (Figure 10Q–T).

### 2.11 Targeted Metabolomics Analysis of Arachidonic Acid Pathway in *SLC16A10* Knockdown Keratinocytes

**Figure 11:**
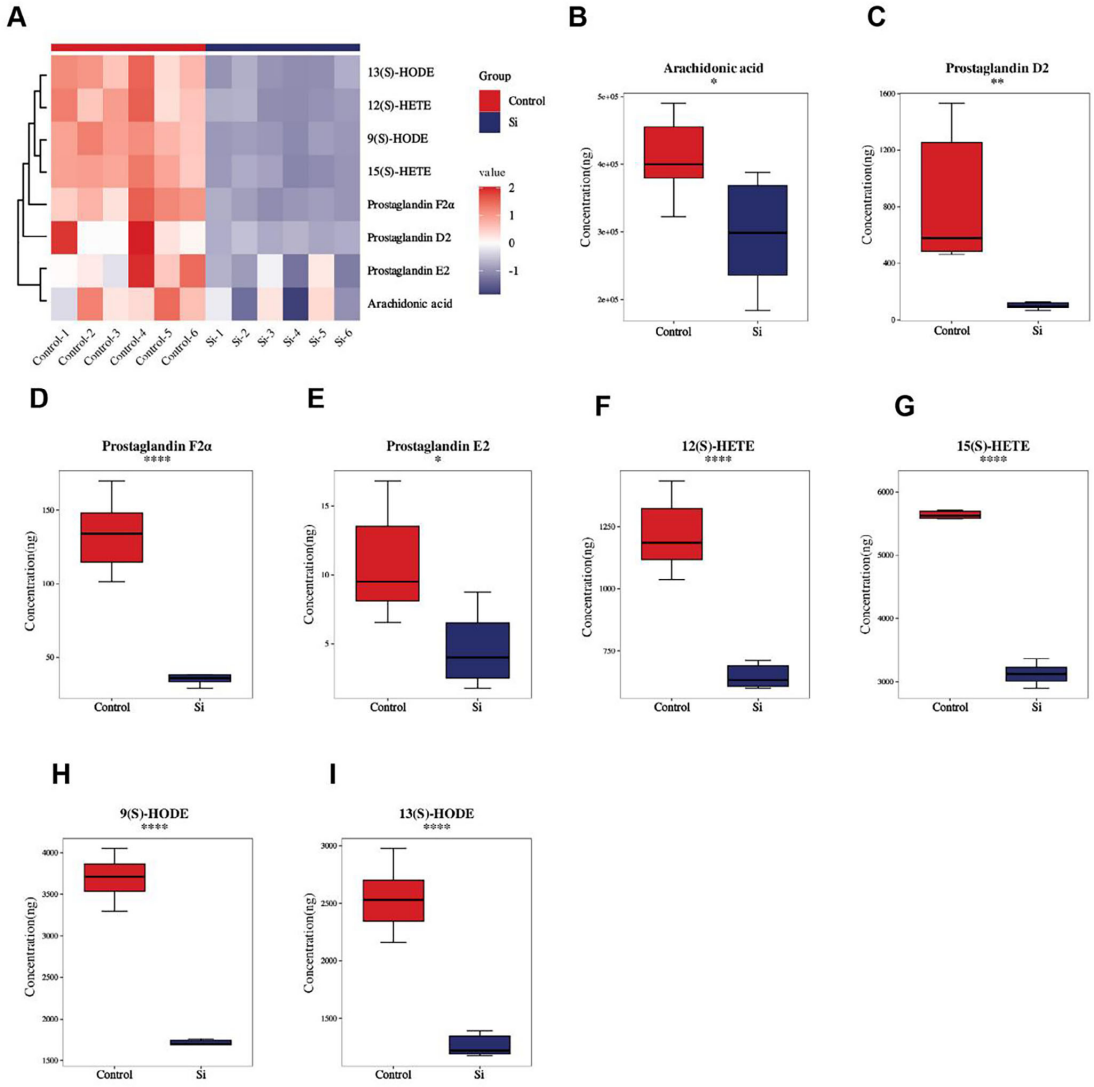
Targeted metabolomics analysis of AA pathway metabolites in *SLC16A10* knockdown keratinocytes. A) Heatmap showing relative concentrations of AA pathway metabolites in control and *SLC16A10* knockdown keratinocytes. Each column represents an individual biological replicate. The color scale represents log2‐transformed values, with red indicating higher concentrations and blue indicating lower concentrations. B–I) Arachidonic acid, PGD2, PGF2α, PGE2, 12(S)‐HETE, 15(S)‐HETE, 9(S)‐HODE, 13(S)‐HODE concentrations measured by targeted metabolomics, n = 6. Data are presented as box plots showing median, quartiles, and range. Statistical significance was determined by unpaired t‐test: ^*^ *P* < 0.05, ^**^ *P* < 0.01, ^***^ *P* < 0.001, ^****^ *P* < 0.0001. Control, scrambled siRNA control; Si, *SLC16A10* siRNA knockdown; AA, arachidonic acid.

To further investigate the regulatory role of SLC16A10 in AA metabolism, we performed targeted metabolomics analysis of AA metabolic pathway‐related metabolites using an in vitro keratinocyte model with *SLC16A10* knockdown (**Figure** **11**; Figure S7 and Table S6, Supporting Information). The results demonstrated that compared to the control group, intracellular AA levels were significantly reduced in the *SLC16A10* knockdown group (Figure 11B), indicating that *SLC16A10* downregulation can modulate AA metabolic homeostasis. Further analysis of pro‐inflammatory metabolites derived from AA revealed significant reductions in prostaglandin series metabolites (Figure 11C–E; Table S6, Supporting Information), with prostaglandin D2 (PGD2) showing the most pronounced decrease, while prostaglandin F2α (PGF2α) and prostaglandin E2 (PGE2) were reduced to 27% and 43% of control levels, respectively, and 8‐iso‐prostaglandin F2α (8‐iso‐PGF2α) was dramatically decreased to 8% of control levels. Additionally, inflammation‐related metabolites in the lipoxygenase (LOX) pathway, including 12(S)‐HETE and 15(S)‐HETE(Figure 11F,G), were significantly diminished in the *SLC16A10* knockdown group, while linoleic acid metabolites 9(S)‐HODE and 13(S)‐HODE (Figure 11H,I) were substantially reduced, and leukotriene B4 (LTB4) (Table S6, Supporting Information) showed a declining trend without reaching statistical significance (*P* = 0.0917). Collectively, these targeted metabolomics findings demonstrate that *SLC16A10* downregulation not only reduces AA levels per se but also significantly suppresses the biosynthesis of downstream pro‐inflammatory metabolites across multiple metabolic pathways, particularly prostaglandin series and hydroxy fatty acid metabolites.

## 3 Discussion

Metabolic disorders contribute significantly to the pathogenesis of psoriasis.^[^ ^4^, ^11^, ^15^ ^]^ Current immunotherapy strategies have limitations, necessitating further research on metabolism‐targeted therapies for psoriasis.^[^ ^9^, ^10^ ^]^ In this study, we identified and investigated the role of a key biomarker, *SLC16A10*, in the pathogenesis of psoriasis.

Our MR analysis, combined with LASSO regression, identified three biomarkers: *SLC16A10*, *IP6K2*, and *ITPR3*. All three biomarkers exhibited significant diagnostic value; however, only *SLC16A10* expression was consistent with the MR results, exhibiting notable potential research value. We identified *SLC16A10* as a risk factor for psoriasis (OR > 1). Notably, it also exhibited a causal relationship with guttate psoriasis according to the MR analysis, with a significantly increased risk (OR > 2). Guttate psoriasis is a subtype of psoriasis vulgaris and is closely related to plaque psoriasis.^[^ ^10^, ^23^ ^]^ Although several studies have demonstrated differences between the two subtypes, distinguishing them based on the circulating levels of IFN‐γ and interleukins in the blood remains challenging.^[^ ^24^, ^25^ ^]^ While targeting the IL‐23/IL‐17 axis exerts a therapeutic effect on guttate psoriasis, most existing immune‐targeted therapies, such as IL‐17 or IL‐23 inhibitors, remain ineffective against this condition.^[^ ^23^, ^26^, ^27^ ^]^ The notable difference observed in this study in the ORs of *SLC16A10* between psoriasis and guttate psoriasis suggests its role in distinguishing guttate psoriasis from plaque psoriasis. Furthermore, GSEA revealed that the biomarkers were enriched in metabolic pathways, such as nucleotide, lipid, and carbohydrate metabolism, which are highly related to the pathogenesis of psoriasis.^[^ ^16^, ^17^ ^]^ Specifically, nucleotide metabolism influences the development of psoriasis.^[^ ^4^ ^]^ IP6K2 plays a role in the early stages of glucose‐induced cytokinesis, generating 5‐IP7, a kinase that regulates biological processes, including cell growth and apoptosis, metabolic homeostasis, and immunity.^[^ ^28^ ^]^ ITPR3 mediates intracellular calcium release and plays a crucial role in animal growth by regulating energy metabolism and exocytosis.^[^ ^29^ ^]^ Our GSEA for *IP6K2* and *ITPR3* revealed enrichment in metabolic pathways involved in energy metabolic processes, such as glycolysis. Energy metabolism influences the function of KCs and related immune cells, contributing to the development of psoriasis.^[^ ^1^, ^4^, ^11^ ^]^ Notably, AA metabolism, a pathway associated with *SLC16A10*, exhibited a strong correlation with inflammation. AA serves as an inflammatory mediator that induces vasodilation, and its metabolites regulate vascular tone and participate in immune surveillance.^[^ ^30^ ^]^ In individuals with psoriasis, skin lesions possess a higher concentration of AA metabolites than that in the adjacent non‐lesional skin and the skin of healthy individuals.^[^ ^31^ ^]^ *SLC16A10* is crucial for maintaining the homeostasis of thyroid hormones and aromatic amino acids, and AA metabolites are affected by thyroid hormone status.^[^ ^32^, ^33^ ^]^ Therefore, we speculated that *SLC16A10* may be involved in AA metabolism by influencing thyroid hormone levels, subsequently affecting the pathogenesis of psoriasis by regulating inflammation, glucose metabolism, and immune surveillance.

We performed scRNA‐seq analyses to identify fundamental transcriptomic changes in KCs of individuals with psoriasis. *SLC16A10* expression was significantly upregulated in KCs, whereas it was markedly downregulated in MCs. As key cells in psoriasis pathogenesis, KCs are co‐regulated by various factors, including gene regulation, cytokines, receptors, and transcription factors, which influence the onset and maintenance of psoriasis.^[^ ^1^, ^15^, ^20^ ^]^ Melanocytosis, a key feature of psoriasis, is observed in psoriatic skin lesions and is typically accompanied by epidermal hyperplasia.^[^ ^34^ ^]^ Furthermore, the severity of psoriasis is strongly correlated with circulating amino acid levels, with significant alterations observed in phenylalanine and tyrosine levels in affected individuals.^[^ ^35^ ^]^ Moreover, in response to skin irritation and damage, KCs release cytokines and rapidly activate AA metabolism through the cyclooxygenase and lipoxygenase pathways; however, this activation is transient.^[^ ^36^ ^]^ In the present study, differential analysis of metabolic pathways in KCs and MCs indicated enrichment in amino acid, lipid, and energy metabolism. Notably, AA metabolism was a shared pathway between both cell subpopulations, with the most significant level of activity observed in MCs. As mentioned previously, despite the established relationship between the severity of psoriasis and circulating amino acid levels, the precise relationship between AA metabolism and MCs in psoriasis remains unclear. Notably, melanin production in MCs is stimulated by prostaglandin E2 (PGE2), while PGE2 synthesis is affected by AA levels, suggesting a potential correlation between AA content and melanin production.^[^ ^37^ ^]^ In the present study, the pseudo‐time analysis combined with GSVA revealed that AA metabolism in KCs and MCs was more active in the LS group than in the CTL group, with MCs exhibiting a more complex metabolic condition. Notably, the active pathways in KCs from the LS group were highly consistent with the GSEA results for *SLC16A10*. These results suggest that significant *SLC16A10* upregulation may affect metabolic homeostasis in KCs and MCs participate in the development of psoriasis.

To validate the potential effects of SLC16A10 in psoriasis, we performed comprehensive in vitro and in vivo experiments using human epidermal samples, psoriasis mouse models, KCs, and MCs. Compared to the healthy human epidermis, the epidermis of individuals with psoriasis exhibited increased epidermal thickness, cell proliferation, and inflammatory immune responses. *SLC16A10* was significantly upregulated in the epidermis of individuals with psoriasis, accompanied by a remarkable increase in AA content. In mice with psoriasis, *Slc16a10* knockdown significantly alleviated the severity of psoriasis, as evidenced by a significant decrease in PSI scores, epidermal thickness, and cell proliferation. Moreover, the AA content was reduced by *Slc16a10* knockdown, and excessive inflammatory and immune responses were alleviated. In KCs under inflammatory stimulation, *SLC16A10* was upregulated, and *SLC16A10* silencing resulted in a prominent decrease in the AA content and the expression of several inflammatory indicators. However, we observed opposite trends in inflammation‐stimulated MCs. Specifically, under inflammation induction, MC proliferation was significantly increased, whereas *SLC16A10* expression was markedly downregulated, accompanied by a prominent decrease in melanogenesis and AA content. These experimental results were consistent with our bioinformatics analyses, confirming the important role of *SLC16A10* in the pathogenesis of psoriasis. These data highlight a strong association between SLC16A10 and AA, demonstrating the role of SLC16A10 in promoting the development of psoriasis by regulating thyroid hormone homeostasis. This regulation enhances KC proliferation and the inflammatory response. Our knockout experiments also demonstrated the potential of *SLC16A10* in metabolism‐based treatments for psoriasis. Furthermore, *SLC16A10* may provide broader treatment options than existing immune‐targeted therapy regimens, with the potential of more favorable therapeutic outcomes, particularly in guttate psoriasis.

The proliferation of MCs is proportional to melanin production. However, our experiments revealed an opposite trend. An increased number of MCs is a pathological feature of psoriasis.^[^ ^34^ ^]^ Post‐inflammatory hypopigmentation is a skin abnormality that occurs after the resolution of inflammatory or infectious skin diseases, including psoriasis. It is characterized by a partial or complete loss of skin pigmentation.^[^ ^38^, ^39^ ^]^ Notably, *SLC16A10* expression is proportional to the melanin content in MCs.^[^ ^40^ ^]^ Therefore, our scRNA‐seq and experimental results suggest that SLC16A10, in addition to promoting the development of psoriasis, may contribute to post‐inflammatory hypopigmentation. Further, *SLC16A10* overexpression increases melanin levels in MCs and may play a role in UVB‐induced hyperpigmentation.^[^ ^40^ ^]^ Our experiments demonstrated a positive correlation between *SLC16A10* downregulation and a corresponding decrease in AA levels. Collectively, we hypothesize that SLC16A10 may inhibit PGE2 synthesis in MCs by reducing AA content, which in turn leads to decreased melanogenesis and ultimately results in post‐inflammatory hypopigmentation.

In this study, we hypothesize that SLC16A10 may play a role in melanogenesis, although its precise mechanisms still require experimental validation. SLC16A10 could modulate melanin synthesis by influencing intracellular metabolic dynamics and signaling cascades. Our initial findings suggest that SLC16A10 expression correlates with energy metabolism in melanocytes, particularly in glucose and fatty acid utilization pathways. For instance, the transporter activity of SLC16A10 may alter the accumulation of key metabolites, thereby shaping the biochemical microenvironment essential for melanin production. Moreover, MITF, the central transcriptional regulator of melanocyte function, directly governs the expression of melanogenic genes.^[^ ^41^, ^42^ ^]^ We speculate that SLC16A10 may interact with the MITF pathway, potentially influencing MITF expression or activity, which is essential for melanin synthesis. While direct experimental confirmation is still lacking, the proposed involvement of SLC16A10 in melanogenesis merits deeper investigation. Elucidating these mechanisms could advance our understanding of pigment biology and reveal novel therapeutic targets for dermatological disorders such as psoriasis.

Very importantly, the identification of SLC16A10 as a metabolic driver in psoriasis pathogenesis raises the translational question of its druggability. However, as of now, there are limited resources available for direct inhibitors targeting SLC16A10. Existing databases, such as the MedChemExpress (MCE) and Sigma, primarily list siRNA sequences and antibodies, but no specific small molecule inhibitors targeting SLC16A10 have been identified. The development of SLC16A10 inhibitors presents a promising therapeutic avenue for psoriasis, particularly given the significant role of SLC16A10 in regulating AA metabolism and its association with inflammatory responses in keratinocytes. To advance this development, a multi‐faceted approach is essential. First, utilizing structural biology techniques, such as cryo‐electron microscopy and X‐ray crystallography, will provide insights into the 3D structure of SLC16A10, enabling structure‐based drug design. Molecular docking studies can then screen potential small molecules for effective binding, while artificial intelligence (AI)‐assisted drug discovery platforms can expedite the identification of novel compounds. Additionally, the potential for drug repurposing should be explored by utilizing databases like DrugBank and the Open Targets Platform to identify existing medications with off‐target effects on SLC16A10, capitalizing on their established safety profiles. Once promising candidates are identified, rigorous preclinical evaluations, including in vitro studies in keratinocyte models followed by in vivo assessments in psoriasis animal models, will be crucial to confirm therapeutic potential and minimize adverse effects. Collaborative efforts between academia and the pharmaceutical industry will further enhance access to advanced technologies and resources. While challenges remain, it is essential to foster innovation in the development of effective SLC16A10‐targeted therapies for psoriasis and other inflammatory conditions.

Our study has some limitations that warrant further consideration. First, the MR data originated from blood samples, whereas the samples used in the subsequent analyses were all obtained from skin tissues. The MR results revealed *IP6K2* and *ITPR3* as risk factors for psoriasis; however, their gene expression in disease samples was lower than that in healthy samples. This finding may be attributed to differences in the source of the samples. Second, the correlation between *SLC16A10* and guttate psoriasis has not been fully verified owing to limitations in data and experimental samples. Moreover, the role of *SLC16A10* in MC remains unclear. Therefore, to address these limitations, appropriate datasets should be collated to validate the roles of biomarkers in the pathogenesis of psoriasis.

In conclusion, we identified and validated a key biomarker, *SLC16A10*, with diagnostic value. Functional enrichment, scRNA‐seq analysis, and in vitro and in vivo experiments demonstrated that *SLC16A10* plays a role in the pathogenesis of psoriasis by participating in AA metabolism. *SLC16A10* knockout or silencing significantly alleviated the severity of psoriasis, suggesting its potential as a therapeutic target for psoriasis. Notably, this biomarker may offer a potential treatment for guttate psoriasis, which does not respond to existing immunotherapies. Additionally, the aberrant *SLC16A10* expression in MCs suggests that *SLC16A10* induces post‐inflammatory hypopigmentation by inhibiting melanogenesis. Our study offers new insights into the pathogenesis of psoriasis by focusing on metabolism and identifying a new diagnostic biomarker and therapeutic target, *SLC16A10*. Our findings provide novel directions for further research, enhancing diagnostic measures and facilitating the development of targeted metabolic therapies for psoriasis.

## 4 Experimental Section

### Collection of Public Datasets

Psoriasis RNA‐seq datasets were retrieved, GSE14905 and GSE13355, and the scRNA‐seq dataset, GSE173706, from the Gene Expression Omnibus (GEO) database.^[^ ^43^ ^]^ GSE14905 contains data on 33 psoriasis lesion skin (LS) samples, 21 healthy individual skin (CTL) samples, and 28 uninvolved skin (NS) samples from individuals with psoriasis. GSE13355 includes data on 58 LS, 64 CTL, and 58 NS samples. GSE173706 includes scRNA‐seq data for 14 LS and 8 CTL samples. A total of 2752 MRGs identified in a previous study were extracted.^[^ ^44^ ^]^ Single‐nucleotide polymorphisms (SNPs) used for the instrumental variable (IV) selection for Mendelian randomization (MR) were obtained from the eQTLGen consortium.^[^ ^45^ ^]^ The outcome used for MR was downloaded from the IEU OpenGWAS database (ID finn‐b‐L12_PSORIASIS).^[^ ^46^ ^]^ The outcome used for MR validation was obtained from the IEU OpenGWAS database, and the IDs of the respective datasets were listed in Table S1 (Supporting Information).

### Identification of Differentially Expressed Genes and DE‐MRGs

Using filtering criteria of *padj* < 0.05 and |log2FoldChange| > 0.5, the limma package (v. 3.58.1) was used to perform differential expression analysis between the LS and CTL groups and obtain differentially expressed genes (DEGs) from GSE14905.^[^ ^47^ ^]^ Volcano plots were generated using the ggplot2 package (v.3.5.0, https://ggplot2.tidyverse.org). A heatmap was created using the ComplexHeatmap package (v.2.18.0) to visualize the expression patterns of the TOP 10 upregulated and downregulated DEGs.^[^ ^48^ ^]^ DE‐MRGs were obtained through the intersection of DEGs and MRGs, and the results were visualized using the ggVennDiagram package (v.1.2.2).^[^ ^49^ ^]^

### Functional Enrichment Analysis

The clusterProfiler package (v.4.4.4) was used to perform enrichment analyses based on gene ontology (GO) and Kyoto Encyclopedia of Genes and Genomes (KEGG) databases.^[^ ^50^ ^]^ The filtering condition was *p*‐value < 0.05. The ggstar (v.1.0.4, https://github.com/xiangpin/ggstar/) and ggplot2 packages were used to visualize the enrichment results.

### MR, Sensitivity Analyses, and MR Validation

In the MR analysis, DE‐MRGs and psoriasis were used as the exposures and outcome, respectively. The screening criteria for instrumental variables (IVs) were: (1) p < 5×10^−8^; (2) R^2^ < 0.001, kb < 10000; (3) directionality test; and (4) F > 10. MR‐PRESSO was applied to remove biased IVs. Subsequently, the *mr()* function in the TwosampleMR package (v.0.5.10) was used in conjunction with 5 algorithms for MR analysis, including MR Egger, weighted median, inverse variance weighted (IVW), simple mode, and weighted mode.^[^ ^51^ ^]^ IVW minimizes the impact of heterogeneity on MR results through a random effects model (REM). It was sensitive to horizontal pleiotropy and yields the most reliable MR estimates.^[^ ^52^ ^]^ Moreover, the *mr_scatter_plot()* function was applied to generate scatter plots showing the correlation between exposure and outcome.

For the heterogeneity test, the *mr_heterogeneity()* function was used, with Q_pval > 0.05 representing no heterogeneity. In cases where heterogeneity was present, the REM of the IVW method was employed to estimate the MR effects. The *mr_pleiotropy_test()* function was applied to perform a horizontal pleiotropy test, and a pval > 0.05 indicated the absence of horizontal pleiotropy. Finally, the *mr_leaveoneout()* function was used for the leave‐one‐out analysis to detect any severely biased SNPs.

Exposures and IVs for MR validation were selected using the same conditions as described previously, with psoriasis and its subtypes representing the outcome (Table S1, Supporting Information). The TwosampleMR package and IVW algorithm were utilized to estimate the MR results for each biomarker, and sensitivity analyses were subsequently performed.

### Identification of Biomarkers and Differential Expression Analysis

A LASSO logistic regression was conducted for significant exposure variables in GSE14905 using the *glmnet* package (v.4.1‐8) with default parameters.^[^ ^53^, ^54^, ^55^ ^]^ Cross‐validation errors were used to filter genes obtained from the results of the *lambda.min* analysis. Moreover, the pROC package (v.1.18.5) was employed to generate receiver operating characteristic (ROC) curves and assess the diagnostic value of the feature genes in GSE14905 and GSE13355.^[^ ^56^ ^]^ The ggpubr package (v.0.4.0, https://rpkgs.datanovia.com/ggpubr/) was used to analyze the differential expression of biomarkers in GSE14905 and GSE13355.

### Gene Set Enrichment Analysis for Biomarkers

Gene set enrichment analysis (GSEA) for biomarkers obtained from the KEGG enrichment analysis was performed using the clusterProfiler package with *padj* < 0.05 and |NES| > 1 as the filtering criteria. The GseaVis package (v.0.0.5; https://github.com/junjunlab/GseaVis) was used to visualize the results. The GOSemSim package (v.2.28.1) was used for functional similarity analysis and visualization.^[^ ^57^ ^]^ Spearman's correlation analysis for these biomarkers was conducted, and scatter plots were generated using the ggExtra package (v.0.10.1, https://github.com/daattali/ggExtra).

### Data Preprocessing for Single‐cell RNA‐seq (ScRNA‐Seq)

The *CreateSeuratObject()* function in the Seurat package (v.5.0.1) was used to filter single‐cell RNA‐seq (scRNA‐seq) data from the GSE173706 dataset, with quality control parameters set to a minimum of 3 cells and 200 features.^[^ ^58^ ^]^ The original dataset did not contain information regarding sequencing depth or cell capture efficiency. Prior to quality control, it was observed that the proportion of mitochondrial genes in the cells was predominantly below 10%. This low mitochondrial proportion (<10%) indicates minimal RNA leakage due to cell damage, suggesting that the cells were viable. The distribution of nCount and nFeature was relatively concentrated, indicating low technical noise, with a high correlation of 0.93 between nCount and nFeature. This strong correlation suggested that the sequencing saturation was sufficient. Based on the data, the estimated sequencing depth was ≈50 K reads per cell, which aligns with the standards set by 10x Genomics. Mitochondrial genes were obtained using the *PercentageFeatureSet()* function, which retained cells with a proportion of mitochondrial genes less than 10%, yielding 16,006 genes and 31,350 cells. The *NormalizeData()* was used for global scaling normalization. The *FindVariableFeatures()* functions were used to identify genes with high variation in expression between cells, selecting 2000 highly variable genes using the “vst” method. Subsequently, a principal component analysis was performed, and the top 30 principal components were selected for subsequent cluster analysis.

### Cell Clustering and Annotation

The *FindNeighbors()* and *FindClusters()* functions in the Seurat package were used for unsupervised cluster analysis, and the results were visualized using t‐SNE. Moreover, the *FindAllMarkers()* function was used to identify positive marker genes, with the parameters set as follows: min.pct = 0.6, only.pos = TRUE, and logfc.threshold = 0.5. Marker gene expression was estimated using the Wilcox method. Subsequently, the marker genes for each cell subpopulation were determined using the CellMarker database and based on a previous study.^[^ ^59^ ^]^ The t‐distributed stochastic neighbor embedding (t‐SNE) clustering map was used to visualize the distribution of cell subpopulations within the overall cell subpopulations and between the LS and CTL groups.

### Biomarker Expression and Gene Set Variation Analysis

Dot plots were used to demonstrate the differential expression of biomarkers among cell subpopulations. Gene set variation analysis (GSVA) was performed for key cells using the GSVA package (v.1.50.0), and significant differential KEGG metabolism‐related pathways between the LS and CTL groups in key cells were obtained using the limma package (*padj* < 0.05). Results were visualized using the *pheatmap()* function.

### Pseudo‐Time Analysis

The Monocle package (v.2.28.0) was used for pseudo‐time analysis of key cells.^[^ ^60^ ^]^ Cell density curves were obtained using the ggplot2 package. Based on the KEGG gene set, the pathway score was calculated using the GSVA package and visualized using the *pheatmap()* function.

### Human Samples

Human skin samples were obtained from individuals with psoriasis via punch biopsy under local anesthesia and from healthy donors during plastic surgery. All participants provided written informed consent. The study adhered to the principles of the Declaration of Helsinki and was approved by the Research Ethics Board of the Air Force Medical Center of the PLA (approval number 2024‐135‐S01).

### Animals

*Slc16a10* heterozygous (*Slc16a10* ^+/−^) mice with the C57BL/6J background were obtained from VIEWSOLID BIOTECH (Beijing, China) and maintained under specific pathogen‐free conditions. Mice were housed in groups of three per cage under a regular 12‐h light/dark cycle, temperature range of 22 ± 1 °C, and relative humidity of 40–70%. Six‐week‐old mice were shaved and treated daily with 50 mg 5% imiquimod (IMQ) (Aldara, 3 M Pharmaceuticals) applied to their dorsal skin to induce psoriasiform hyperplasia.

All mice were classified into four groups: IMQ‐, IMQ‐ *Slc16a10* ^+/−^, IMQ+, and IMQ+ *Slc16a10* ^+/−^. The psoriasis severity index (PSI) was used to assess the severity of skin inflammation on the back and shoulders of the animals, as previously described.^[^ ^61^ ^]^

Erythema, scaling, and skin thickness were key indicators of the PSI, each of which was evaluated on a discrete scale of 0 to 4: 0 (none), 1 (slight), 2 (moderate), 3 (marked), and 4 (very marked). The total PSI was calculated by summing the scores of all indicators, ranging from 0 to 12 for each mouse. Skin tissues were collected after 5 or 8 days of treatment. To minimize suffering, mice were euthanized via CO_2_ inhalation. The experiments adhered to the National Institutes of Health guidelines for laboratory animal care and were approved by the Ethics Committee of the Laboratory Animal Center, Air Force Medical Center of the PLA (approval number 2024‐039‐S01).

### Cell Culture and Transfection

KC cell lines (HaCaT) (cat. CTCC‐002‐0012) and human primary melanocytes (MCs) (cat. CTCC‐118‐HUM) were purchased from MeisenCTCC (Zhejiang, China) and grown in customized media (MeisenCTCC, CTCC‐002‐0012‐CM and CTCC‐002‐0012‐CM, respectively). Cells were transfected with siRNAs targeting *Slc16a10* using Lipofectamine 3000 (Invitrogen) for gene knockdown experiments. siRNAs (cat. AM16708) were purchased from Thermo Fisher Scientific (Waltham, MA, USA). An in vitro psoriasis‐like model was established using a protocol adapted from a previous study.^[^ ^62^ ^]^ HaCaT cells and MCs were plated at appropriate densities and allowed to adhere for 12 h. The cells were then exposed to a cytokine cocktail, M5, comprising 5 inflammatory mediators in equal concentrations (1:1:1:1:1 ratio): IL‐17A (cat.200‐17; PeproTech, Cranbury, NJ, USA), IL‐22 (cat.200‐22; PeproTech), IL‐1α (PeproTech, cat.200‐01A), oncostatin M (cat.300‐10H; PeproTech), and tumor necrosis factor‐alpha (TNF‐α; cat.300‐01A; PeproTech). The M5 cytokine mixture was applied at concentrations of 5, 10, and 15 ng/mL for each cytokine. The stimulation period lasted 24–48 h, following which the cells were harvested for experimentation. The control group was maintained in parallel and treated with phosphate‐buffered saline (PBS) instead of the cytokine mixture. KCs and MCs were classified into four groups depending on stimulation with M5 or silencing of *SLC16A10*: M5‐, M5‐ siSLC16A10, M5+, and M5+ siSLC16A10.

### Histological and Immunohistochemical Assays

Paraffin‐embedded skin specimens of 4‐µm thickness were sectioned and stained with hematoxylin and eosin (HE). For immunohistochemical staining, the sections were blocked with 5% bovine serum albumin and incubated with primary antibodies against Ki67 (1:800, cat.9449; cat. 12202; Cell Signaling Technology [CST], Danvers, MA, USA), CD4 (1:200, cat.25229; cat. 93518; CST), F4/80 (1:500, cat. 70076; CST), and CD45 (1:200, cat. 70257; CST) overnight at 4 °C. Following primary antibody incubation, sections were incubated with horseradish peroxidase‐conjugated secondary antibodies. Finally, the slides were digitally scanned using a Slide Scanner (KFBIO, Ningbo, China). Images were acquired using the K‐Viewer software (v.1.5.3.1, KFBIO, Ningbo, China). Epidermal thickness and quantitative analysis of Ki67, CD4, F4/80, and CD45 expression were performed using the Fiji software (v.2.9.0).

### Immunofluorescence Staining

Skin sections were incubated with primary antibodies against *SLC16A10* (1:500; cat. ab121519; Abcam, Cambridge, UK) overnight at 4 °C. Following PBS washes, sections were incubated for 1 h with Cy3‐conjugated secondary antibodies. Subsequently, the nuclei were counterstained with DAPI. The stained sections were digitally scanned using a Slide Scanner (3DHISTECH, Budapest, Hungary). Images were acquired using the CaseViewer software (v.2.4).

### Western Blotting

For western blotting (WB) analysis, protein samples from cells or tissues were separated using SDS‐PAGE on 10%–12% gels, transferred onto polyvinylidene fluoride (PVDF) membranes, and probed with primary antibodies against the following proteins: SLC16A10 (1:1000, cat. PA5‐50760; Invitrogen, Carlsbad, CA, USA), β‐actin (1:5000, cat.4 970S; CST), PLA2 (1:500, cat. 22030‐1‐AP; Proteintech, Rosemont, IL, USA), COX‐2 (1:1000, 12375‐1‐AP, Proteintech, Rosemont, IL, USA) and NF‐κB (1:1000, 10745‐1‐AP, Proteintech, Rosemont, IL, USA). Subsequently, the membranes were incubated with horseradish peroxidase‐conjugated secondary antibodies at a 1:5000 dilution. Protein bands were visualized using an ECL kit and detected using a ChemiDoc MP imaging system (Bio‐Rad Laboratories, Hercules, CA, USA). Western blot quantification was performed using Fiji software, and protein levels were normalized against β‐actin expression.

### Real‐Time Quantitative Polymerase Chain Reaction

Total RNA from cells and tissues was extracted using the MolPureCell/Tissue total RNA kit (cat. 19221ES60, Yeasen, China) following the manufacturer's protocol. Real‐time quantitative polymerase chain reaction (RT‐qPCR) was performed using the PrimeScript RT Reagent Kit after assessing the purity of the extracted RNA. mRNA expression was quantified using the SYBR mixture on the Mx3000P real‐time PCR system (Agilent Technologies, Santa Clara, CA, USA). Amplification differences between samples and controls were calculated using the Ct (ΔΔCt) method and normalized against *GAPDH* or *Β‐ACTIN* expression. The primers used for RT‐qPCR are listed in Table S2 (Supporting Information).

### Arachidonic Acid Level Detection

Arachidonic acid (AA) levels in the cells and tissues were quantified using an enzyme‐linked immunosorbent assay (ELISA) kit for AA (E‐EL‐0051, Elabscience Biotechnology Inc., Wuhan, China) following the manufacturer's protocol.

### Statistical Analysis

Statistical analyses were performed using the GraphPad Prism software (version 8.0.1; GraphPad Inc., La Jolla, CA, USA). Data were assessed for normality and homogeneity of variance. Normally distributed data are expressed as mean ± standard deviation (SD), and non‐normally distributed data are presented as medians with interquartile ranges (IQRs). For multiple group comparisons, one‐way analysis of variance (ANOVA) followed by Tukey's post‐hoc test was employed for normally distributed data with homogeneous variances. In cases of normally distributed data with heterogeneous variances, Brown–Forsythe and Welch ANOVA were used, followed by Holm–Sidak's multiple comparisons test. Non‐normally distributed data were analyzed using the non‐parametric Kruskal–Wallis test, followed by Dunn's multiple comparisons test. Two‐way repeated measure ANOVA was used to analyze two factors experiments. For two‐sample comparisons, unpaired t‐tests were used. For all analyses, statistical significance was set at *P* < 0.05.

### Ethical Statement

This study was approved by the Research Ethics Board of the Air Force Medical Center of the PLA (approval number 2024‐135‐S01). All individuals who provided tissue samples signed the informed consent form. The experiments adhered to the National Institutes of Health guidelines for laboratory animal care and were approved by the Ethics Committee of the Laboratory Animal Center, Air Force Medical Center of the PLA (approval number 2024‐039‐S01).

## Conflict of Interest

The authors declare no conflict of interest.

## Author Contributions

J.Y., Y.C., B.L., and S.W. contributed equally to this work. H. C. supervised the project and obtained the funding. X.‐k. J. and W. L. provided critical feedback on the manuscript. X. Y., Y. C., and X. L. collected the public data used in this study and designed the in vitro and in vivo experiments. H. L. performed bioinformatics analyses. X. Y., X. L., P. S., G. G., T. L., Y. J., and H. L. performed in vitro and in vivo experiments. M. B., J. X., and Z. L. assisted in data interpretation and experimental validation. J. Y., Y. C., and B. L. wrote the manuscript with input from all the authors. All authors reviewed and approved the final version of the manuscript.

## Supporting information



Supporting Information

Supporting Tables

## Data Availability

The data that support the findings of this study are available in the supplementary material of this article.
