# Supplementary material for: Role of SLC16A10 in Psoriasis Through the Regulation of Arachidonic Acid Metabolism in Keratinocytes
Source: Adv Sci (Weinh). 2025 Sep 8;12(39):e17093. doi: 10.1002/advs.202417093 (PMC12533357; doi:10.1002/advs.202417093)
Supplement: Supplementary file 1 — Supporting Information [file ADVS-12-e17093-s002.docx]

# **Supporting Information**

Role of SLC16A10 in Psoriasis Through the Regulation of Arachidonic Acid Metabolism in Keratinocytes

*Jingyuan Yang, Yixuan Chen, Bowei Li, Shuang Wu, Hang Liang, Xingyue Yang, Xiaozhen Li, Ping Sun, Guangjin Guo, Ting Li, Yuying Jia, Haijiao Li, Mengqi Bai, Jie Xu, Zhijie Liu, Wei Liu*, Xiangkang Jiang*, Hong Cai**

# **Supplementary experimental section**

**Supplementary Method 1. Cell Culture and Construction of Stable Cell Lines**

A lentiviral-based shRNA approach was utilized to implement three distinct shRNA sequences targeting *SLC16A10*. These shRNA sequences were cloned into a lentiviral vector to facilitate the knockdown of *SLC16A10* expression. Simultaneously, a full-length *SLC16A10* coding sequence was synthesized and cloned into a lentiviral overexpression vector to achieve *SLC16A10* overexpression in HaCaT cells. To package the lentivirus, 293T cells were used. HaCaT cells were seeded in 6-well plates at 60-70% confluence, after which the lentiviral constructs were added to the cells in the presence of polybrene (8 μg/mL) to enhance viral transduction. The cells were incubated with the virus for 12-16 h, after which the medium was replaced with fresh culture medium. Following a 48-h infection period, puromycin was employed to select for successful transduction. The effects of *SLC16A10* knockdown and overexpression were subsequently confirmed through Western blot and RT-qPCR analyses. The resulting stable cell lines were then utilized for downstream functional assays. The specific sequences for SLC16A10 knockdown and overexpression in this study are detailed in **Table S5**.

**Supplementary Method 2. Enzyme-linked Immunosorbent Assay (ELISA)**

The expressions of IL-6 (E-EL-H6156, Elabscience Biotechnology Inc., Wuhan, China) and TNF-α (E-EL-H0109, Elabscience) in cells were measured using a commercially available enzyme assay according to the manufacturer’s protocol.

# **Supplementary Method 3. T_3_ Uptake Assay**

To determine the kinetic characteristics of T_3_ uptake in HaCaT cells, a time course experiment was conducted. Forty-eight hours post-transfection, the cells were washed with Dulbecco’s Phosphate Buffered Saline (D-PBS) and incubated at 37 °C for 10 to 60 min with 150,000 counts per minute (cpm) of T_3_ labeled with ^125^I ([^125^I]-T_3_ PerkinElmer) in D-PBS supplemented with 0.1% BSA. After the incubation period, the cells were rapidly washed twice with ice-cold assay buffer and subsequently lysed using 0.1 M NaOH. The cell-associated radioactivity was quantified by counting the cell lysates using a γ-counter, and the results were expressed in cpm.

**Supplementary Method 4. Targeted Metabolomics Analysis**

In the in vitro keratinocyte culture model following M5 treatment, the supernatant was discarded, and the cell pellets from the *SLC16A10* knockdown and control groups were resuspended in 99 % methanol, rapidly frozen in liquid nitrogen, and stored at −80 °C. The samples were prepared and analyzed on an LC/MS platform by Berry Genomics (Beijing, China). In short, Analyses were performed using an UHPLC (1290 Infinity LC, Agilent Technologies) coupled to a QTRAP (AB Sciex 6500+). the mobile phase contained A = 0.1% FA in water and B = 0.1% FA in ACN. The samples were in the automatic sampler at 4 °C, and the column temperatures were kept constant at 35 °C. The gradients were at a flow rate of 400 µL/min, and a 2 µL aliquot of each sample was injected. The gradient was 30% B was kept for 1.0min, and increased to 90% in 1.0-9.0min, and kept for 9.0-11.0min, then B was reduced to 20% in 11.0-11.1min and kept for 11.1-14.0 min. The QC samples were used for testing and evaluating the stability and repeatability of this system, at the same time, setting the standard mixture of metabolites, used for the correction of chromatographic retention time. In ESI negative modes, the conditions were set as follows: source temperature 500 °C, ion Source Gas1 (Gas1): 50, Ion Source Gas2 (Gas2): 50, Curtain gas (CUR): 30, ionSapary Voltage Floating (ISVF)-4500 V; Adopt the MRM mode detection ion pair. The Multiquant software was used to extract chromatographic peak area and retention time. Use the standards correct retention time, to identify the metabolites. Data analysis, including principal component analysis and hierarchical clustering, was performed using R.

# **Supplementary Figures**

**
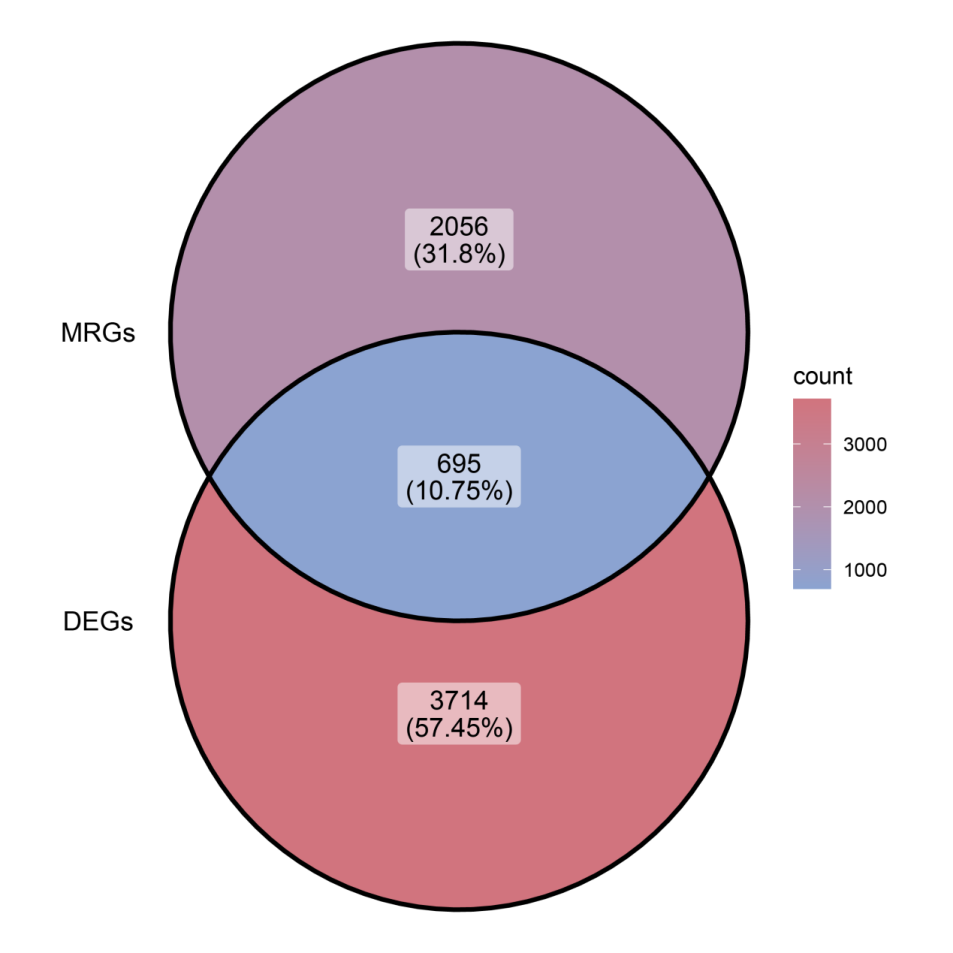
**

# **Figure S1. Venn diagram of DE-MRGs.**

**
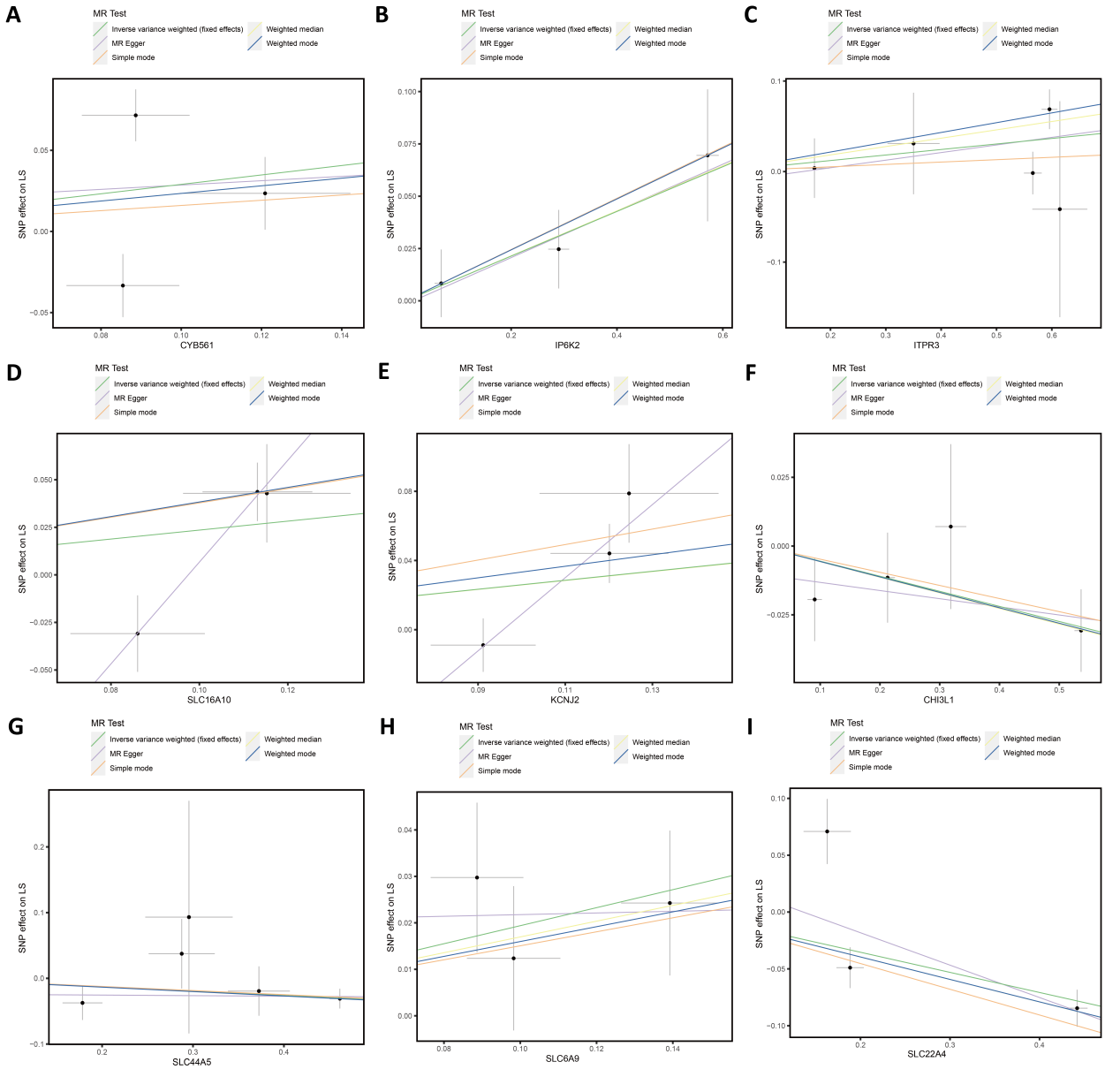
**

**Figure S2**. Scatter plots of MR fitting results on (A) *CYB561*; (B) *IP6K2*; (C) *ITPR3*; (D) *SLC16A10*; (E) *KCNJ2*; (F) *CHI3L1*; (G) *SLC44A5*; (H) *SLC6A9* and (I) *SLC22A4.* Note: Each dot on the scatter plot represents a SNP, and a positive slope of the line indicates a risk factor, while a negative slope indicates a safety factor. When an intercept is present, the presence of confounding factors is implied.

**
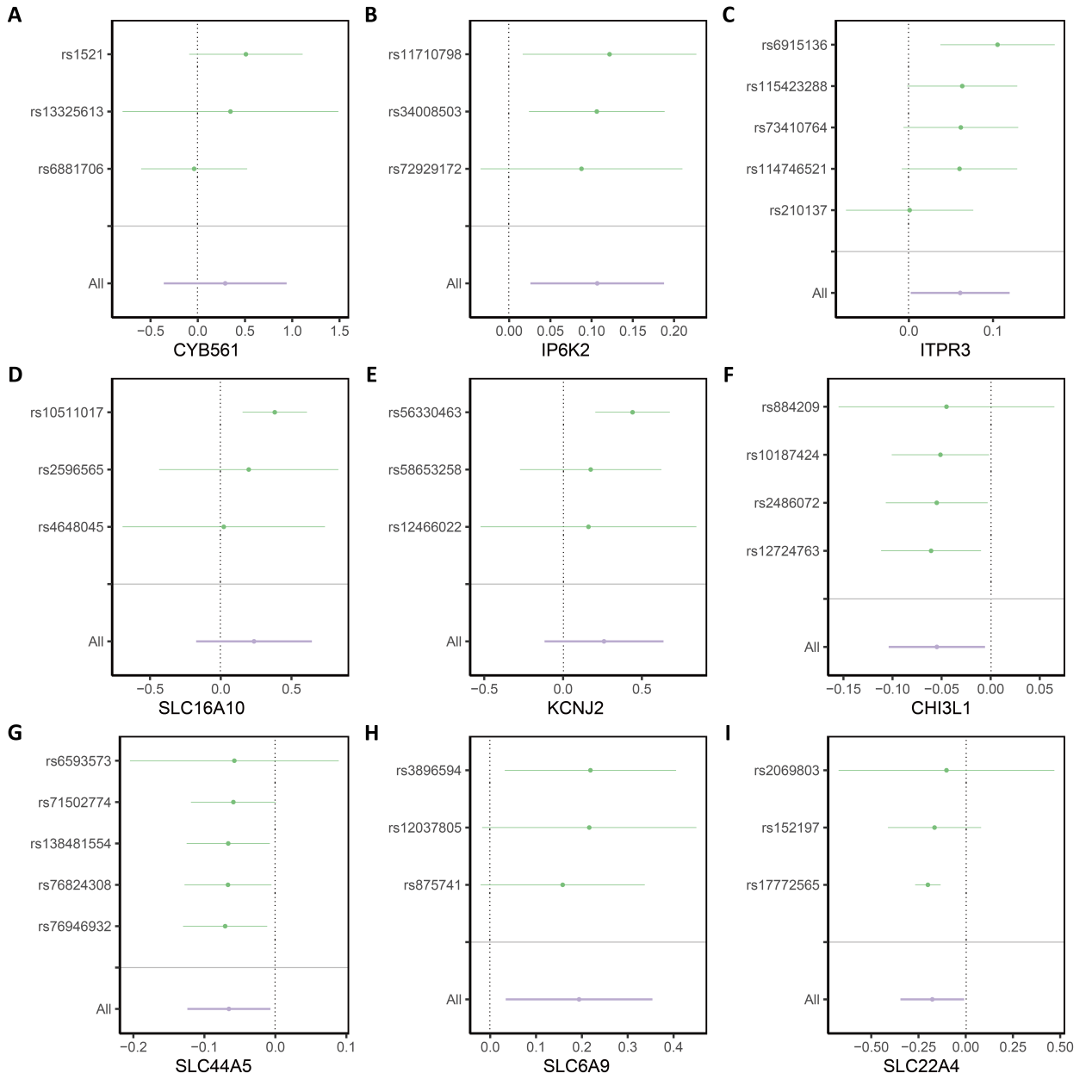
**

**Figure S3**. The forest plot of Leave-one-out analysis of MR on (A) *CYB561*; (B) *IP6K2*; (C) *ITPR3*; (D) *SLC16A10*; (E) *KCNJ2*; (F) *CHI3L1*; (G) *SLC44A5*; (H) *SLC6A9* and (I) *SLC22A4.*

**
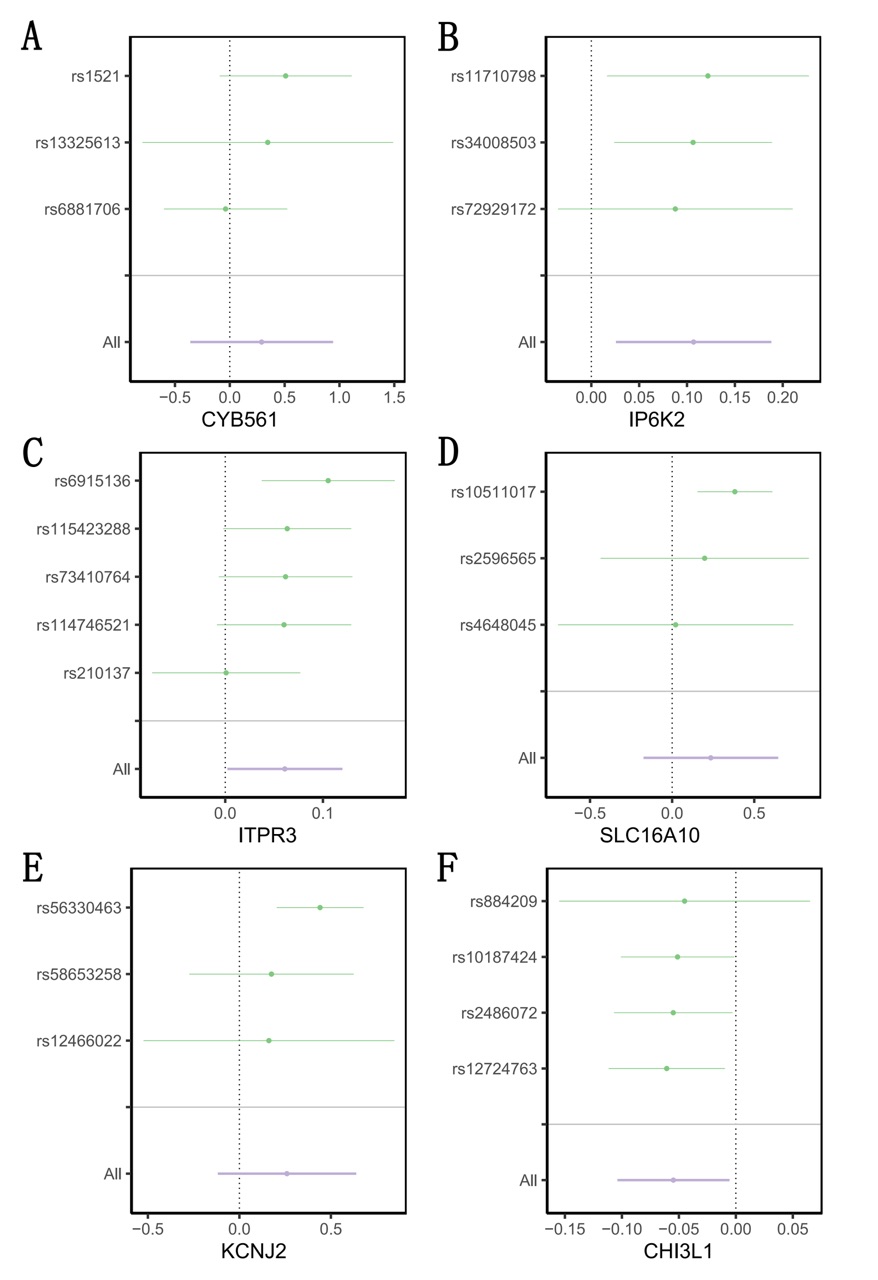
**

**Figure S4.** The forest plot of Leave-one-out analysis in validation MR. (A) The forest plot of Leave-one-out analysis in MR of psoriasis vulgaris on *CYB561*; (B) The forest plot of Leave-one-out analysis in MR of psoriasis vulgaris on *IP6K2*; (C) The forest plot of Leave-one-out analysis in MR of arthropathic psoriasis on *ITPR3*; (D) The forest plot of Leave-one-out analysis in MR of unspecified psoriasis on *SLC16A10*; (E) The forest plot of Leave-one-out analysis in MR of strict psoriasis vulgaris on *KCNJ2*; (F) The forest plot of Leave-one-out analysis in MR of guttate psoriasis on *CHI3L1*.

*
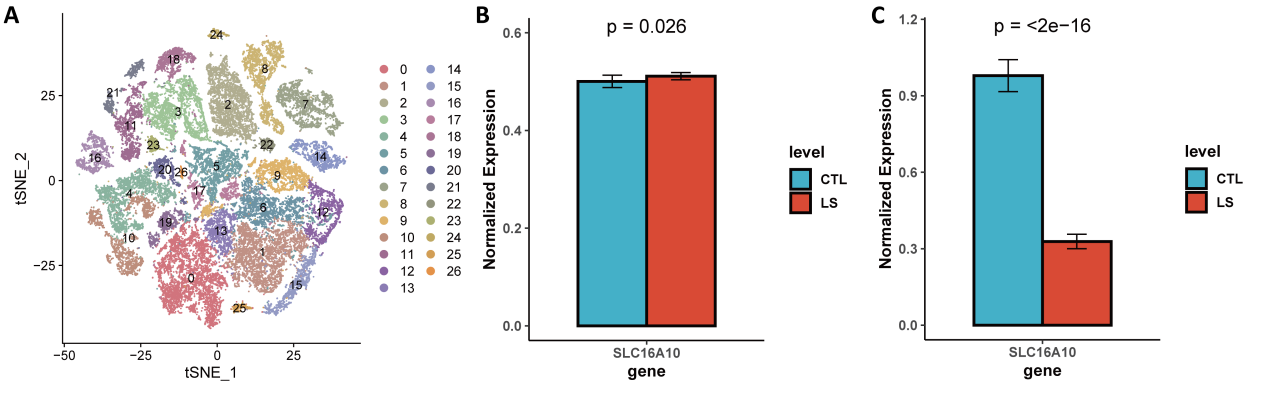
*

**Figure S5**. Single-cell RNA-seq analysis results. (A) tSNE of cell clusters from unsupervised clustering at a default resolution; (B) Histogram of differential expression of *SLC16A10* in KCs; (C) Histogram of differential expression of *SLC16A10* in MCs. MCs, melanocytes; KCs, keratinocytes.


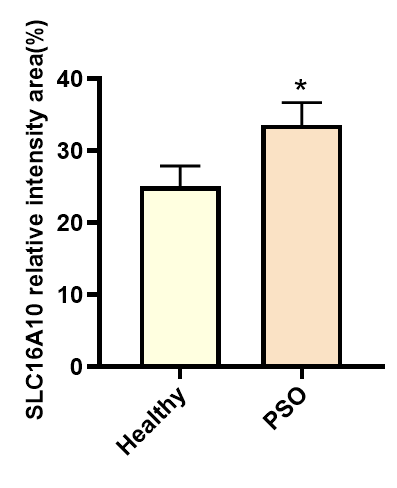


**Figure S6**. The statistics of SLC16A10 intensity area; n = 6, ^*^*P* ＜ 0.05, compared with healthy group.


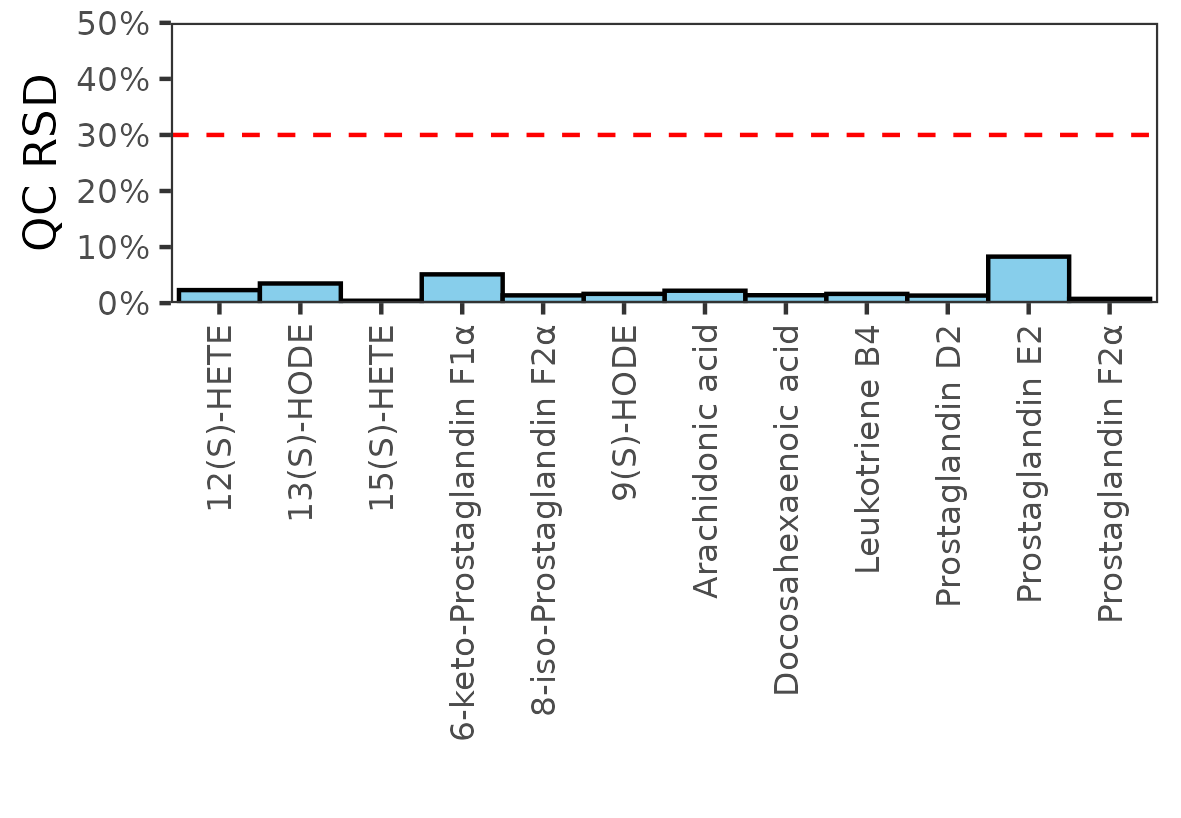


**Figure S7.** Quality control assessment of targeted metabolomics analysis. QC RSD (relative standard deviation) values for each metabolite are shown, with the red dashed line indicating the 30% threshold for analytical precision. All metabolites demonstrated QC RSD values below 30%, confirming the reliability and reproducibility of the analytical method.
